# RAD sequencing resolves fine-scale population structure in a benthic invertebrate: implications for understanding phenotypic plasticity

**DOI:** 10.1098/rsos.160548

**Published:** 2017-02-08

**Authors:** David L. J. Vendrami, Luca Telesca, Hannah Weigand, Martina Weiss, Katie Fawcett, Katrin Lehman, M. S. Clark, Florian Leese, Carrie McMinn, Heather Moore, Joseph I. Hoffman

**Affiliations:** 1 Department of Animal Behavior, University of Bielefeld, Postfach 100131, 33501 Bielefeld, Germany; 2 Department of Earth Sciences, University of Cambridge, Downing Street, Cambridge, Cambridgeshire, CB2 3EQ, UK; 3 Faculty of Biology, Aquatic Ecosystem Research, University of Duisburg-Essen, Universitaetsstrasse 5, 45141 Essen, Germany; 4 British Antarctic Survey, Natural Environment Research Council, High Cross, Madingley Road, Cambridge CB3 0ET, UK; 5 Agri-Food and Biosciences Institute, Fisheries and Aquatic Ecosystems, 18a Newforge Lane, Belfast BT9 5PX, UK

**Keywords:** *Pecten maximus*, phenotypic plasticity, microsatellite, single nucleotide polymorphism, morphometrics, great scallop

## Abstract

The field of molecular ecology is transitioning from the use of small panels of classical genetic markers such as microsatellites to much larger panels of single nucleotide polymorphisms (SNPs) generated by approaches like RAD sequencing. However, few empirical studies have directly compared the ability of these methods to resolve population structure. This could have implications for understanding phenotypic plasticity, as many previous studies of natural populations may have lacked the power to detect genetic differences, especially over micro-geographic scales. We therefore compared the ability of microsatellites and RAD sequencing to resolve fine-scale population structure in a commercially important benthic invertebrate by genotyping great scallops (*Pecten maximus*) from nine populations around Northern Ireland at 13 microsatellites and 10 539 SNPs. The shells were then subjected to morphometric and colour analysis in order to compare patterns of phenotypic and genetic variation. We found that RAD sequencing was superior at resolving population structure, yielding higher *F*_st_ values and support for two distinct genetic clusters, whereas only one cluster could be detected in a Bayesian analysis of the microsatellite dataset. Furthermore, appreciable phenotypic variation was observed in size-independent shell shape and coloration, including among localities that could not be distinguished from one another genetically, providing support for the notion that these traits are phenotypically plastic. Taken together, our results suggest that RAD sequencing is a powerful approach for studying population structure and phenotypic plasticity in natural populations.

## 1. Introduction

Studies that characterize the genetic structure of natural populations can provide important insights into the processes of gene flow, genetic drift and selection [1]. For example, the presence of genetically distinct populations can reveal the influence of sometimes unforeseen barriers to gene flow [2], whereas a lack of structure can point towards unexpected dispersal mechanisms, such as the rafting of eggs on seaweed or driftwood (e.g. [3–7]). Population genetic studies have also been widely used to test for phenotypic plasticity [3,8–10]. These studies typically explore the extent to which phenotypic differences among populations relate to the underlying population genetic structure, following the logic that phenotypic differences among genetically unstructured populations should reflect the influence of environmental variation.

To study population structure and its implications for phenotypic plasticity, particularly over fine spatial scales where population structure may be weak, it would be desirable to genotype as many genetic markers as possible. Although markers like amplified fragment length polymorphisms have sometimes been used because they do not require prior genomic information, microsatellites have long been the marker of choice as they are co-dominant, tend to have high allelic diversity and are relatively straightforward to genotype. However, they are also time-consuming to develop and impractical to genotype in large numbers, limiting most studies to the use of around 10–20 markers. Fortunately, recent advances in high-throughput sequencing technologies and the development of genotyping-by-sequencing approaches [11] such as restriction site-associated DNA (RAD) sequencing [12] now make it possible to genotype thousands of single nucleotide polymorphisms (SNPs) in virtually any species. Although SNPs are individually less informative than microsatellites, simulations suggest that thousands of SNPs should outperform tens of microsatellites for uncovering population structure [13]. This is supported by recent empirical studies showing that RAD sequencing is capable of resolving fine-scale population structure (e.g. [14,15]) although, to our knowledge, only Rašić *et al*. [16] directly compared empirical estimates of population structure obtained from microsatellites and RAD sequencing. They found that the population structure of yellow fever mosquitoes (*Aedes aegypti*) sampled from three different continents was better resolved with 18 147 SNPs than eight microsatellites.

Marine invertebrates are particularly interesting from the perspective of population structure for a number of reasons. Owing to the excellent dispersal capabilities of many of these species and a perceived absence of barriers to gene flow, marine invertebrate populations were for many years assumed to be demographically open [17]. However, this view has been increasingly brought into question with the emergence of genetic studies uncovering often unexpected patterns of population structure [18,19]. These studies have revealed interactions between life history and the physical environment [20] and also uncovered effects of microhabitat specificity and spatio-temporal variability on population structure (e.g. [21]). Population structure has even been discovered in broadcast spawning species with larvae that can persist in the water column for several weeks or months (e.g. [22]), suggesting that stretches of deep water and oceanic currents may represent significant barriers to gene flow to most if not all marine invertebrate species.

The great scallop *Pecten maximus* (also known as the king scallop) is a benthic mollusc that occurs along much of the Northeastern Atlantic coastline. This species is predominantly sedentary, although to escape predators, it can swim short distances by rapidly bringing the valves together, which expels a jet of water on either side of the dorsal hinge [23]. Fertilization takes place in the water column after spawning and the larvae are planktonic for three to eight weeks before metamorphosing and settling as spat [24]. As secondary dispersal via byssus drifting has also been reported in this species [25], great scallops are expected to be capable of dispersing considerable distances.

Consistent with knowledge of the life history of this species, previous genetic studies variously using allozymes, mitochondrial DNA and random amplified fragment polymorphisms have revealed very little in the way of population structure within the British Isles or France, with the exception of a single commercially cultured population at Mulroy Bay in Northern Ireland [26–28]. More recently, a larger pan-European study found evidence for two genetically distinct populations, one in Norway and the other comprising a number of Atlantic coastal populations sampled along a latitudinal gradient from Ireland to Spain [29]. However, it remains to be seen whether genetic methods that offer greater resolution such as RAD sequencing will uncover weaker, fine-scale population structuring.

Another motivation for studying great scallops is their commercial importance, with annual global capture production being in excess of 75 000 tons (http://www.fao.org/fishery/species/3516/en) and the most important commercial fisheries being around France and the British Isles [30]. Despite this, a successful large-scale scallop aquaculture industry has failed to materialize in Europe, largely due to the inconsistent supply of wild or hatchery seed, low survivorship and a long production cycle [31]. Many areas where scallops are harvested, such as the Northern Irish fishery, have also shown gradual declines in the numbers and biomass of scallops landed, which is resulting in longer fishing trips [31]. Consequently, there is considerable interest both in understanding geographical patterns of variability in phenotypic traits such as growth rate [32] and in evaluating whether artificial stock enhancement could help to restore wild scallop stocks.

An ideal opportunity to study the fine-scale population structure of *P. maximus* in relation to phenotypic variation is provided by a long-term research programme on Northern Irish great scallop populations carried out by the Agri-Food Bioscience Institute (AFBI) on behalf of the Department of Agriculture, Environment and Rural Affairs (DAERA). Annual surveys have been conducted around the coast of Northern Ireland since 1992, with a number of additional sites having been incorporated into the survey scheme in 2011 and 2012 based on stakeholder engagement and the analysis of onboard vessel monitoring systems. The main aim of these surveys is to monitor the impact of commercial scallop fishing activity on naturally occurring great scallop populations by quantifying variability in the abundance of scallops as well as by-caught species over space and time. These surveys provide important information in support of the scientific assessment and management of Northern Irish scallop fisheries.

During the 2015 survey, scallops were collected from nine sites around the coast of Northern Ireland. By genotyping these at 13 microsatellites and subjecting a representative subset of the samples to ddRAD sequencing [33], a variant of RAD sequencing, the pattern and strength of population structure found at the two markers could be directly compared. Digitized shell outlines were also subjected to geometric morphometric analysis, and the intensity and extent of brown pigmentation on the inner shell surface were quantified, allowing us to test the hypothesis that shell shape and coloration are phenotypically plastic.

## 2. Material and methods

### 2.1. Sample collection and DNA extraction

**Figure 1.**
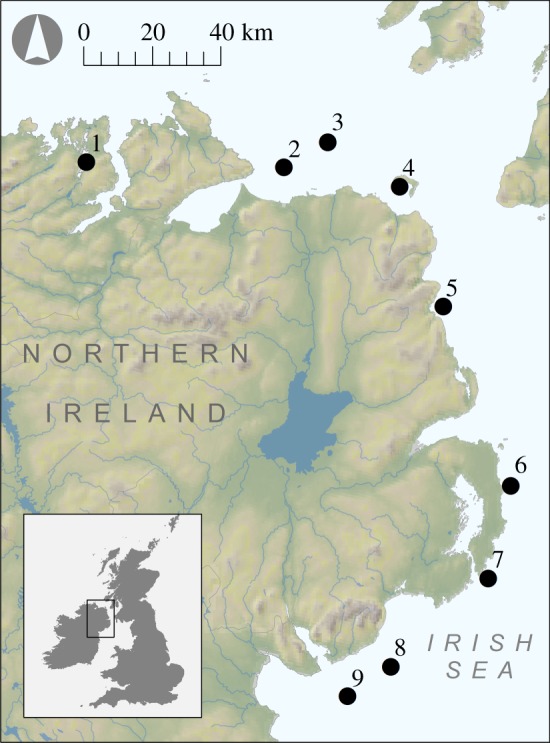
Map showing the study area with sample collection locations.

Twenty scallop samples were collected from each of nine different locations along the northeastern coast of Northern Ireland (figure 1) between 9 and 19 February 2015. Scallops from location one (Mulroy Bay) are cultured from locally sourced broodstock, whereas scallops from locations two to nine are naturally occurring. The latter were collected by four dredges attached to a beam, with each dredge having 10 mm teeth spaced 75 mm apart and belly ring size of 85 mm. A fine mesh liner (less than 10 mm) was placed inside one of the dredges to retain juvenile scallops and smaller associated fauna. From each adult scallop, approximately 1 cm^3^ of adductor muscle was taken for genetic analysis and stored in 95% ethanol at −20°C. The shells were cleaned, rinsed with Milli-Q water and retained for morphometric analysis. Whole genomic DNA was extracted following an adapted phenol-chloroform protocol [34] and DNA concentrations were quantified using a Qubit Fluorometer (Life Technologies).

### 2.2. Microsatellite genotyping

All of the samples were genotyped at 15 previously developed microsatellite loci (see electronic supplementary material, table S1 for references). These were PCR amplified in four separate multiplexed reactions using a Type It Kit (Qiagen) as described in electronic supplementary material, table S1. The following PCR profile was used: one cycle of 5 min at 95°C; 28 cycles of 30 s at 94°C, 90 s at the specified annealing temperature (Ta)°C and 30 s at 72°C; and one final elongation step of 15 min at 60°C (see electronic supplementary material, table S1 for Ta). Fluorescently labelled PCR products were then resolved by electrophoresis on an ABI 3730xl capillary sequencer and allele sizes were scored automatically using GeneMarker v. 2.6.2 (http://www.softgenetics.com/GeneMarker.php). To ensure high genotype quality, all traces were manually inspected and any incorrect calls were adjusted accordingly.

### 2.3. RAD sequencing

Five samples from each locality were used to prepare a ddRAD library [33] consisting of 45 samples pooled together, according to the protocol of Schweyen *et al*. [35] with some modifications: the double digestion was conducted with FastDigest enzymes NsiI and MspI (Thermo Scientific) in a 45 µl reaction volume using 800 ng RNase-digested DNA. In the ligation, P7 adapters with small modifications relative to the set of Schweyen *et al*. [35] were used (electronic supplementary material, information 1). First, the Degenerate Base Region (DBR) sequence, which is used to detect and remove PCR duplicates, was modified to enable a better hybridization. Second, the overhang was adjusted to fit to the overhang produced by MspI. Third, an inline barcode similar to the index barcode was added. The size selection following the adapter ligation was conducted using SPRIselect beads (Beckman Coulter). Therefore, 45 µl of SPRIselect were mixed with 50 µl of sample (ratio of 0.9) and a left-side size selection was conducted following the manufacturer's protocol. The samples were eluted with 28 µl of sterile water. The resulting PCR products were purified using the AmpureXP bead system (Beckman Coulter) with the manufacturer's protocol. Samples were eluted with 28 µl of sterile water and directly used for a dual size selection with the SPRIselect beads following the manufacturer's protocol. In a first step, the right-side size selection, 15.4 µl SPRIselect were mixed with the 28 µl sample (ratio of 0.55). In the following left-side size selection, 8.4 µl of SPRIselect were added leading to a ratio of 0.85. The DNA was eluted using 28 µl of sterile water. After pooling, a final size selection was conducted using the LabChip XTe (PerkinElmer) for the size range of 308 to 462 bp with the DNA 750 assay kit. A detailed protocol can be found in electronic supplementary material, information 2. The final libraries were sequenced on two lanes of an Illumina HiSeq 2000 sequencer using 100 bp paired-end reads.

The resulting sequence data were analysed using a custom bioinformatic workflow as described below. First, trimming, quality control, identification of PCR duplicates and demultiplexing of raw reads were performed using the program trimmomatric [36] and the scripts ‘double_indexing.pl’ and ‘preprocess_radtags.pl’ [35], which are available at https://github.com/evoeco/radtools. Then, processing of the cleaned data was carried out using Stacks v. 1.35 [37]: denovo_map.pl was used to assemble tags and rxstacks was then utilized to increase the quality of the called genotypes. Subsequently, a whitelist of loci was generated in order to retain only biallelic SNPs genotyped in at least 40 individuals. Where more than one SNP was present in a given tag, only the SNP that was genotyped in the largest number of individuals was retained. All individual genotypes with a stack depth smaller than five were then removed.

### 2.4. Generation of summary statistics

Genepop on the Web [38,39] was used to test each microsatellite locus for deviations from Hardy–Weinberg and linkage equilibria. The dememorization number was set to 10 000, the number of batches to 1000 and the number of iterations per batch to 10 000. The resulting *p*-values were corrected table-wide for the false discovery rate (FDR) [40] using the program *Q*-value [41]. Arlequin v. 3.5.2.2 [42] was also used to calculate the number of alleles plus observed and expected heterozygosities.

### 2.5. Analysis of population structure

To formally test for the presence of population structure, pairwise *F*_st_ [43] values were generated for the microsatellite and RAD datasets. This analysis was implemented in Arlequin with statistical significance being determined based on 1000 permutations of the dataset. We also tested whether population genetic structure could be detected without the need for *a priori* geographical information by conducting a Bayesian cluster analysis of the nuclear marker datasets using Structure v. 2.3.3 [44]. This program employs a Bayesian iterative approach to cluster individual genotypes into *K* populations by subdividing the dataset in a way that maximizes Hardy–Weinberg and linkage equilibrium within the resulting clusters. The membership of each individual in a population is then estimated as *q*, which varies between 0 and 1, with the latter indicating full population membership. Five simulations were run for each value of *K* ranging from one to nine with the burn-in period and Markov chain Monte Carlo repetitions set respectively to 10^5^ and 10^6^. The most likely number of clusters was evaluated using the maximal average value of Ln *P*(*D*), a model choice criterion that estimates the posterior probability of the data. Finally, as structure can struggle to detect genetic differences between populations when *F*_st_ is very low [45], we additionally conducted principle component analyses (PCA) of the microsatellite and RAD datasets using Adgenet v. 2.0.1 [46,47].

### 2.6. Power analysis

To determine the power or the microsatellite and RAD datasets to resolve population structure, we carried out a power analysis using POWSIM v. 4.1 [48]. This program allows the user to adjust a number of user-defined parameters. To reflect our sampling design, we set the number of subpopulations to nine, with 20 samples per subpopulation for microsatellites and five for SNPs. We set the effective population size of the subpopulations to 1000, 2000 and 3000, and we allowed 10 and 20 generations respectively, since population separation. We carried out 500 simulations per analysis and set the number of burn-ins, batches and iterations per batch to 1000, 100 and 1000, respectively.

### 2.7. Morphometric analysis

A geometric morphometric approach based on elliptic Fourier analysis [49,50] was used to characterize shell shape variation both within and between populations. This approach uses Fourier transformations to extract geometric information from complex outlines, described as periodic functions, by decomposing them into the harmonic sum of simple trigonometric functions [51,52]. Low-frequency harmonics approximate coarse-scale variation in the outlines, whereas higher-frequency harmonics capture fine-scale variation [53].

Digital images of the lateral view of left valves were acquired using a high-resolution digital camera (Nikon D3300). Prior to outline digitization, the photographs were processed using Adobe Photoshop, centred and vertically aligned along the hinge axis. Outlines (black masks on a white background) for each of the lateral views were obtained and only the shapes of intact shells (*n* = 180) were retained for further analysis. An algorithm implemented by Claude [52] was used to convert each outline into a list of pixel (*x*, *y*) coordinates sampled along the shell perimeter (electronic supplementary material, figure S1), which were then used as input data for subsequent analyses.

Prior to elliptic Fourier transformation, an outline adjustment through geometric operations on coordinates was performed in order to avoid any normalization bias on shapes prone to bad alignment among their first-fitted ellipses (round and bilaterally symmetric). Therefore, outlines were smoothed to remove any digitization noise, centred, rescaled and aligned using a Procrustes superimposition [54] on 1000 sampled pseudo-landmarks. Shapes invariant to size, rotation and independent of outline position were then obtained [51]. Elliptic Fourier transformation was then computed on the resulting coordinates without normalization. After a preliminary calibration, the first 15 harmonics were selected to encompass 98% of the harmonic power [55]. Four coefficients per harmonic (60 descriptors) were then calculated for each individual and used as variables quantifying the geometric information contained within the outlines [51,52].

The extracted harmonic coefficients were subjected to PCA using a singular value decomposition method in order to visualize the morphological variability among individuals. The first 15 principal components (PCs) which accounted for 95% of the outline variability were considered as new ‘shape’ variables. Multivariate Analyses of Variance (MANOVA) were performed on these 15 PCs to test for a significant effect of population of origin on shape. To visualize differences between populations at the extremes of the morphospace, deformation grids [56] were obtained through formalization of thin-plate spline analysis (TPS, [57]).

### 2.8. Colour analysis

For each individual, we quantified the intensity and extent of brown pigmentation on the inner surface of the left valve. Digital photographs were processed with Adobe Photoshop and ImageJ and then converted from an RGB to a grey scale format (R + G + B/3) with pixel intensity values ranging from zero to 255. Average grey values for the coloured shell area (0–110 intensity pixels) and the proportion of the shell that was coloured were then calculated for each individual. These data were in turn used to calculate a colour index, defined as the square root of the product of mean pigment intensity and the proportion of coloured surface, as shown below

$$\text{colour}\;\text{index}=\sqrt{\frac{\text{average}\;\text{grey}\;\text{value}\times \text{coloured}\;\text{area}}{\text{total}\;\text{shell}\;\text{area}}}.$$

This index provides a measure of the amount of colour on the shells that reflects both the pigment intensity and the proportion of coloured surface. High values indicate shells with high colour intensity and extent, whereas small values correspond to shells with relatively little and weak pigmentation. General Linear Models (GLM: model I ANOVAs) were constructed to test for differences in colour index values among populations. All of the morphological and colour analyses were performed in the R environment [58] and Momocs v. 1.0 [53]. Computer code and accompanying documentation for these analyses are provided as a PDF file written in R markdown (electronic supplementary material, information S3).

## 3. Results

To compare the ability of microsatellites and RAD sequencing to resolve fine-scale population structure, we genotyped 180 great scallops from nine populations around the coast of Northern Ireland at 15 microsatellites, and a subset of 45 individuals comprising five individuals selected at random from each population were also ddRAD sequenced. Genetic variability at the microsatellites was moderately high, with each locus carrying on average 10.9 alleles (electronic supplementary material, table S1). A number of significant deviations from Hardy–Weinberg equilibrium (HWE) were found after table-wide FDR correction (electronic supplementary material, table S1), but most of the microsatellites deviated from HWE in fewer than two populations. However, two of the loci (PmRM027 and PmNH11) were found to deviate from HWE in five and seven populations respectively so we took the conservative measure of excluding them from further analyses.

Illumina sequencing generated 232 797 307 paired-end reads, of which 143 596 562 (61.7%) were retained after filtering based on quality and read length. Of these, a further 42 061 012 reads (29.29%) were removed following the criterion set out in the Material and methods, including 31 033 980 duplicate reads (21.61% of the total number of retained reads). The remaining 101 535 550 reads were assembled into 659 341 tags. After SNP calling and filtering, 10 539 tags were retained for further analysis, each of which contained a single biallelic SNP.

### 3.1. Microsatellites

**Figure 2.**
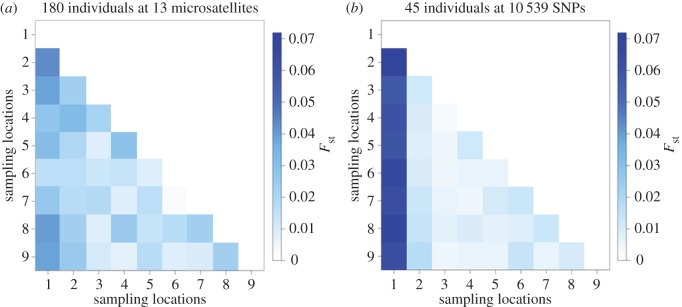
Heatmap depicting pairwise *F*_st_ values calculated using (*a*) 13 microsatellites genotyped in 180 individuals and (*b*) 10 539 SNPs genotyped in 45 individuals.

**Figure 3.**
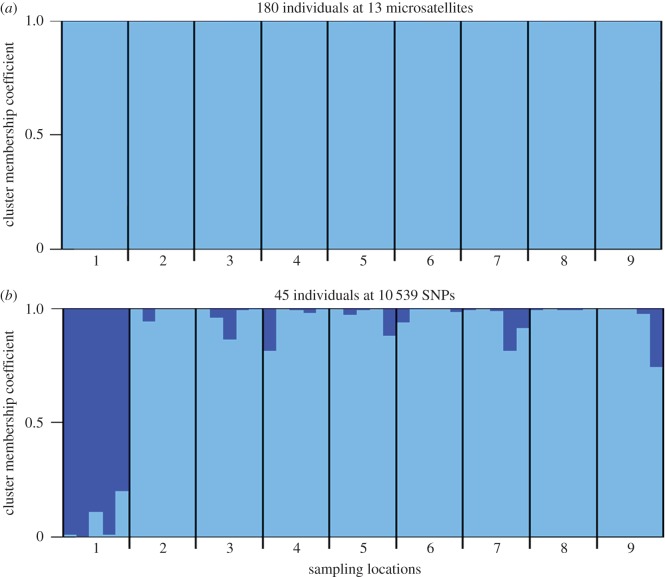
Cluster membership coefficients of (*a*) 180 individuals genotyped at 13 microsatellites; and (*b*) 45 individuals genotyped at 10 539 SNPs. Each individual is represented by a vertical line partitioned into segments of different colour, the lengths of which indicate the posterior probability of membership in each group.

**Figure 4.**
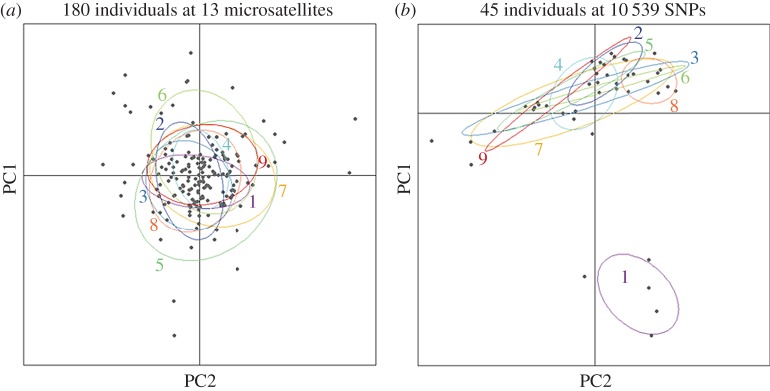
Scatterplot showing individual variation in the first two principal components (PCs) derived from a principal component analysis (PCA) conducted on (*a*) 13 microsatellites genotyped in 180 individuals where PC1 and PC2 explain 2.4 and 2.3% of the genetic variation respectively and (*b*) 10 539 SNPs genotyped in 45 individuals where PC1 and PC2 explain 4.4 and 2.9% of the genetic variation respectively.

To formally test for the presence of population structure, pairwise *F*_st_ values were generated based on 13 microsatellites (figure 2*a*; electronic supplementary material, table S2*a*). The majority of pairwise population comparisons did not yield significant *F*_st_ values after table-wide FDR correction, with the exception of a handful of comparisons involving Mulroy Bay. No association was found between genetic and shortest coastline geographical distance (Mantel's *r* = 0.235, *p* = 0.156). Bayesian cluster analysis of the microsatellite dataset within the program Structure revealed support for a single cluster (*K* = 1, figure 3*a*; electronic supplementary material, figure S2*a*). This suggests that, although many of the pairwise population comparisons involving Mulroy Bay were individually significant, the magnitude of population structure was too low to be detected by Structure. In support of the above analyses, PCA also revealed no evidence for the presence of distinct genetic groups within the microsatellite dataset (figure 4*a*).

### 3.2. Comparison between microsatellites and single nucleotide polymorphisms

To enable a direct comparison between microsatellites and SNPs, five randomly selected individuals per population were genotyped at 10 539 SNPs. Restricting the microsatellite analysis to these 45 individuals, none of the pairwise population comparisons yielded significant *F*_st_ values after table-wide FDR correction (electronic supplementary material, table S3 and figure S3) and Structure found support for a single panmictic population (electronic supplementary material, figure S4). By contrast, the SNP data revealed a number of significant pairwise *F*_st_ values after FDR correction, including the majority of comparisons involving Mulroy Bay and three comparisons among the remaining populations (figure 2*b*; electronic supplementary material, table S2*b*). Notably, the SNP data yielded higher *F*_st_ values than the microsatellite data for comparisons involving Mulroy Bay (figure 2). This was reflected in the outcome of the Structure analysis, which revealed support for the presence of two clusters (*K* = 2, electronic supplementary material, figure S2*b*). Membership coefficients for the two inferred clusters are summarized in figure 3*b*, where each vertical bar represents a different individual and the relative proportions of the two colours indicate the probabilities of belonging to each cluster. Classifying individuals according to their populations of origin, two groups are well defined, the first comprising individuals sampled from Mulroy Bay and the second comprising individuals from the remaining eight populations. Consistent with this finding, PCA revealed a clear difference between scallops from Mulroy Bay and the remaining populations (figure 4*b*).

### 3.3. Power analysis

To determine the power of the microsatellite and SNP datasets to detect population structure, we analysed both datasets using POWSIM (see Material and methods for details). For the microsatellite dataset, power was strongly dependent on the assumed effective population size and time since separation, varying from around 0.37 to 1 (table 1*a*). By contrast, the SNP dataset provided consistently high power for every scenario tested (table 1*b*). This suggests that RAD sequencing provided more than adequate power to detect genetic differences among the populations.

**Table 1.** Results of the power analysis conducted on (*a*) 13 microsatellites and (*b*) 10 539 SNPs; *t* is the time in generations and *Ne* is the effective population size of subpopulations.

(*a*) 13 microsatellites			(*b*) 10 539 SNPs		
*t*	*Ne*	power	*t*	*Ne*	power
10	1000	0.968	10	1000	1
20	1000	1	20	1000	1
10	2000	0.526	10	2000	1
20	2000	0.964	20	2000	1
10	3000	0.376	10	3000	1
20	3000	0.758	20	3000	1

### 3.4. Shell morphology

**Figure 5.**
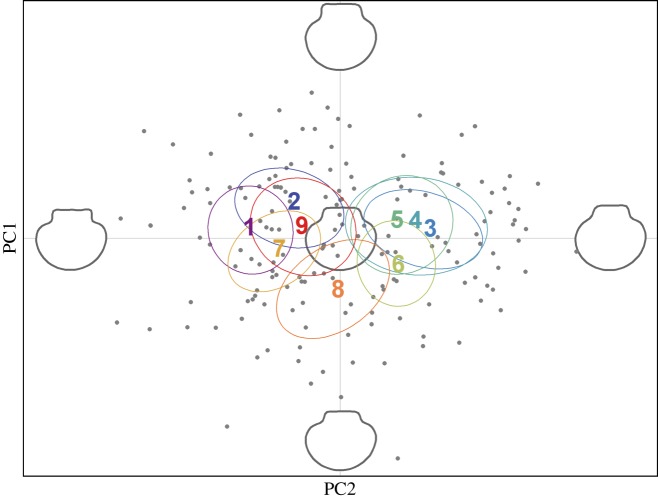
Scatterplot showing individual variation in the first two principal components (PCs) derived from a principal component analysis (PCA) conducted on the elliptic Fourier coefficients of scallop shell shape. PC1 and PC2 explain 38.3 and 18.3% of the variation in shell shape respectively. Extreme and average reconstructed shell outlines are shown in grey.

To analyse shell shape variation, an elliptic Fourier analysis was conducted on the scallop shell outlines. After Fourier decomposition, harmonic coefficients were calculated from the top 15 harmonics, which captured 98% of the shape information, and were used as shape descriptors. PCA was then performed on the extracted coefficients to represent axes capturing most of the morphological variability among the individuals. The first three PCs accounted for 68.1% of the shape variability, and a scatterplot of PC1 and PC2 revealed appreciable variation among the nine populations across the morphospace (figure 5). Specifically, populations 1, 2, 7 and 9, and populations 3, 4, 5 and 6 appeared to form two distinct clusters, while population 8 occupied an intermediate position on the factorial plane.

Next, specific shell features that contributed the most towards the observed pattern of morphological variation were identified. These were represented through the contribution of the first three PCs to the shell outlines for increasing values along the main shape variables (electronic supplementary material, figure S5*a*). PC1 explained 38.3% of the total shape variation and contributed mainly to variation in the angle formed by the shell auricles, with negative values corresponding to relatively square hinges and positive values to more pointed hinges. PC2 explained 18.3% of the total shape variation and contributed mainly to the variation in hinge height, with negative values indicating compact shells with short hinges and positive values indicating elongated shells with well-developed hinges. PC3 explained a further 11.5% of the total shape variation and contributed mainly to the variation in the hinge length, with negative values corresponding to relatively short hinges and positive values to longer hinges.

Multivariate linear models (MANOVAs) based on the top 15 PCs, which captured 95% of the total phenotypic variance, revealed highly significant differences among the populations regardless of whether or not Mulroy Bay was included (all populations: Wilks' *λ* = 0.112, _Approx._*F*_8,161_ = 3.18, *p* < 0.001; excluding Mulroy Bay: Wilks' *λ* = 0.136, _Approx._*F*_7,142_ = 2.89, *p* < 0.001). Thin-plate spline analysis [57] was used to illustrate the main deformation required to pass from one extreme of the morphospace (population one) to the other (population three). The mean shape of population one was characterized by a significantly squarer hinge and rounder shell than population three, which exhibited a more pointed hinge, elongated shape and slight anteroposterior asymmetry of the auricles (electronic supplementary material, figure S5*b*).

### 3.5. Shell colour

**Figure 6.**
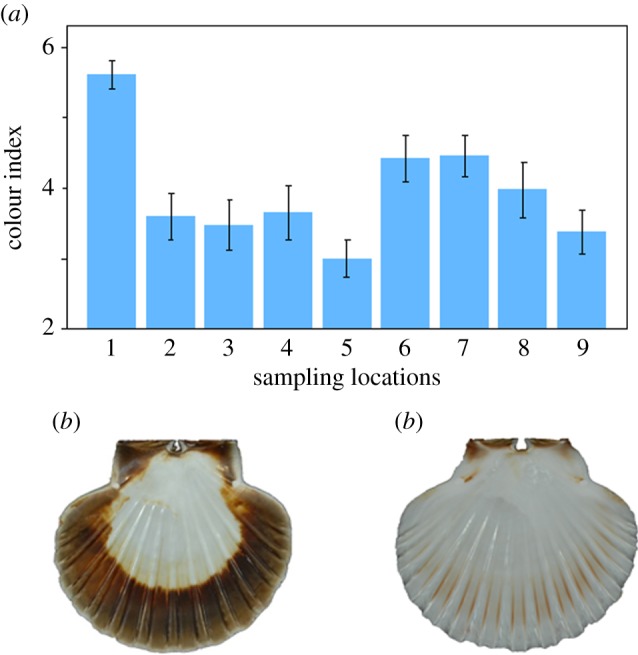
Colour variation among nine scallop populations. Panel (*a*) shows population-specific mean and standard error colour index values. Panels (*b*) and (*c*) show extreme colour phenotypes from Mulroy Bay and population five respectively.

Appreciable variation among individuals was also observed in colour intensity as well as the proportion of the shell that was pigmented. Analysis of variance (ANOVA) revealed highly significant differences in the colour index values of the populations, both overall and after excluding Mulroy Bay (all populations: *F*_8,171_ = 5.61, *p* < 0.001; excluding Mulroy Bay: *F*_7,152_ = 2.59, *p* = 0.015). Geographical variation in the colour index, together with representative phenotypes from Mulroy Bay and population five, is shown in figure 6.

### 3.6. Relationship between genetic and morphometric variation

Finally, we evaluated whether an individual's genotype, represented by the first and second PCs from the PCA of the SNP dataset, was associated with shell shape and coloration, represented by the first PC from the shell outline analysis and the colour index respectively. General linear mixed models (GLMMs) of the two morphological variables were constructed, with population included as a random effect, and the genetic PCs fitted together as predictor variables. Model parameters were estimated using a maximum-likelihood approach in the R package lme4 v. 1.1–12 [59] and *p*-values were derived using *χ*^2^ tests implemented in the R package car v. 2.1–3 [60]. Mulroy Bay was excluded from this analysis due to this population being both genetically and morphologically distinct. No relationship was found between either of the genetic PCs and shell shape (PC1: $\chi_{1}^{2}=1.74$, *p* = 0.187; PC2: $\chi_{1}^{2}=0.15$, *p* = 0.7), suggesting the absence of a link between genotype and shell shape at the individual level (electronic supplementary material, table S4). By contrast, a significant association was found between the first but not the second genetic PC and shell coloration (PC1: $\chi_{1}^{2}=4.4$, *p* = 0.036; PC2: $\chi_{1}^{2}=0.7221$, *p* = 0.395) (electronic supplementary material, table S4). However, caution is warranted as such a weak association could easily result from type I error, given the total number of tests carried out (see Discussion).

## 4. Discussion

Genotyping-by-sequencing approaches like RAD sequencing are expected to outperform small panels of traditional genetic markers for resolving shallow or fine-scale population structure [61], with implications for understanding phenotypic plasticity. However, we are only aware of a single empirical study that directly compared the strength and pattern of population structure resolved by microsatellites and RAD sequencing, and this was over an intercontinental scale [16]. Furthermore, empirical demonstrations of the ability of RAD sequencing to detect relatively subtle fine-scale population structure are only just beginning to emerge (e.g. [14,15]), while, to our knowledge, only one study has used RAD sequencing to investigate phenotypic plasticity [62]. By genotyping nine populations of great scallops from Northern Ireland at 13 microsatellites and 10 539 SNPs, we showed that RAD sequencing is better able to detect fine-scale population structure than a typical sized panel of microsatellites, even with a substantially smaller sample size of individuals. We also uncovered appreciable morphological and colour variation among the populations, despite most of these not being genetically differentiated. A parsimonious explanation for this is that shell shape and coloration both have a plastic component in Northern Irish scallops.

### 4.1. Comparison of microsatellite and RAD sequencing data

Despite SNPs being on average less informative per locus than microsatellites [63], simulations suggest that in very large numbers they have much greater power to detect population structure [13]. Consequently, approaches such as RAD sequencing, which are capable of genotyping many thousands to tens of thousands of SNPs in virtually any organism, are increasingly moving into the mainstream of molecular ecological studies. Although we focused on the fine-scale population structure of *P. maximus*, we believe that our results will be broadly applicable to other species and contexts. First, we used a typical sized panel of microsatellites, which were moderately polymorphic and showed no evidence of systematic deviations from HWE. Second, although the inference of population structure from RAD data can be sensitive to a number of sources of error [64], we took several measures to minimize such errors. Specifically, we used rxstacks to maximize the quality of the called SNPs [65] and also restricted SNP calling to the forward reads, which should minimize the representation of tightly linked loci [66]. Finally, in the wet laboratory we implemented the protocol of Schweyen *et al*. [35] to allow PCR duplicates to be filtered out of the dataset. A remarkable 21.61% of the reads were identified as PCR duplicates and removed from further analyses, suggesting that we effectively eliminated what has been described as a key source of error in ddRAD datasets [67].

In addition to the above, we also carried out a sensitivity analysis of our results to three main parameters that can be adjusted within the denovo_map.pl script in Stacks: -m, -M and -n. -m defines the minimum number of identical raw reads required to create a stack, -M specifies the number of mismatches between loci within individuals and -n specifies the number of mismatches allowed between loci among individuals. We found that although there was minor variation among the datasets generated using different values of -m, -M and -n in the numbers of tags present, observed heterozygosity, average depth of coverage and the total number of SNPs called after quality filtering (electronic supplementary material, table S5), the inference of population structure from these datasets was qualitatively very similar. This is illustrated by electronic supplementary material, figure S6, which shows that *F*_st_ heatmaps based on these alternative SNP datasets reveal very strong similarity to our original results.

To formally compare the strength and pattern of population structure at microsatellites and SNPs, we first calculated pairwise *F* statistics and determined their statistical significance after FDR correction. Even with only five individuals per population genotyped at the SNPs and 20 individuals per population genotyped at the microsatellites, the former yielded a larger number of significant pairwise *F*_st_ values, while after restricting the microsatellite data to the same individuals genotyped for SNPs, we did not find any significant pairwise *F*_st_ values. Moreover, consistently larger *F*_st_ values were obtained for the SNPs in comparison to the microsatellites. A similar result was found by Coates *et al*. [68], who attributed this to homoplasy at the microsatellites.

Arguably, the most powerful tests of population structure need not rely on data on the sampling locations of individuals. We therefore additionally evaluated the ability of the two marker panels to detect population structure using Bayesian cluster analysis. The results were very clear-cut, with Structure finding no evidence of population structure either with the full or the restricted microsatellite datasets, whereas two distinct genetic clusters were identified from the SNP data. Consistent with the pairwise *F*_st_ values, samples from Mulroy Bay formed a distinct genetic cluster with little admixture between this population and the remaining ones. This finding was also supported by PCA, which revealed an absence of any discernible population structure for the microsatellite dataset and a clear separation of Mulroy Bay from the remaining populations for the SNP dataset. Furthermore, our power analysis indicated that while 13 microsatellites had variable and often low power to detect genetic differences between populations, 10 539 SNPs had consistently very high power regardless of the parameters assumed.

Elsewhere, Rašić *et al*. [16] compared the strength of global population structure in the mosquito *Aedes aegypti* measured using eight microsatellites and a large panel of SNPs. They similarly found that population structure could be more clearly resolved with SNPs, and that SNPs yielded higher and more significant pairwise *F*_st_ values than microsatellites. Our study builds upon this empirical foundation by confirming these previous findings in a very different, dispersal-restricted marine species, as well as by showing that RAD sequencing of as few as five samples can resolve population structure over a much smaller geographical scale (tens rather than thousands of kilometres).

Although the signal of population structure is clearly stronger for the SNPs, both sets of markers suggest that Mulroy Bay is genetically the most distinctive of the populations we sampled from along the coastline of Northern Ireland. One reason for this could be that Mulroy Bay is a sea loch with a relatively narrow connection to the open sea that could restrict gene flow to the other coastal populations. However, great scallops are also commercially bottom cultured at Mulroy Bay, which could potentially lead to changes in allele frequencies [69] even if scallop production is mainly based on wild-caught spat. Regardless of the exact explanation, our results are consistent with previous studies based on mitochondrial DNA and random amplified fragment polymorphisms that also found genetic differences between Mulroy Bay and other sites [26–28]. Interestingly, Mulroy Bay is an important population in the sense that it has been repeatedly used as a source of spat for scallop aquaculture both in Ireland and more widely across Europe [70].

### 4.2. Phenotypic variation

Geometric morphometric and shell colour analysis revealed appreciable phenotypic variation among the nine scallop populations sampled. Consistent with the results of the genetic analysis, scallops from Mulroy Bay were phenotypically the most different, being characterized by a squarer hinge shape as well as a greater extent and intensity of brown pigmentation on the inner surface of the left valve. However, highly significant differences in both shell shape and coloration persisted even after excluding Mulroy Bay. By implication, although commercial culturing may be partly responsible for phenotypic differences between scallops from Mulroy Bay and the other populations, natural variation also appears to be present.

Phenotypic differences between the scallops from Mulroy Bay and the other eight populations are confounded by genetic differences, preventing us from drawing firm conclusions as to whether these differences are genetic or plastic. This may reflect the fact that culturing can alter both the genetic composition of a population and the local environment. However, among the remaining eight scallop populations, we found appreciable phenotypic variation but little in the way of genetic differentiation, with only three out of 28 pairwise comparisons yielding significant *F*_st_ values after FDR correction and Structure finding no evidence for subdivision when the analysis was restricted to these populations (electronic supplementary material, figure S7). This apparent lack of a relationship between genotype and phenotype at the population level is consistent with previous studies showing that other traits such as growth rate, body size and crystal microstructure and strength show considerable plasticity in response to environmental heterogeneity in scallops, albeit over a much larger geographical scale [32], as well as to wild and cultured growth conditions over smaller scales [71,72].

To provide further insights into phenotypic plasticity, we conducted additional analyses at the level of the individual, testing for associations between the first and second genetic PCs and shell shape and coloration while including population as a random effect to control for non-independence within populations. No associations were found with shell shape, lending further support to the hypothesis that this trait is phenotypically plastic. By contrast, a weakly significant association was found between the first genetic PC and the colour index. While this provides an interesting avenue for follow-up work, we would caution against interpreting such an association too literally, as the *p*-value was only marginally significant and we cannot therefore rule out a type I error. Furthermore, brown pigmentation on the internal surfaces of shells is known to be a consequence of organic matrix and crystalline microstructure alterations due to calcification abnormalities, which develop over time in scallops [72,73]. These abnormalities are thought to be related to parasitic or bacterial infection and more generally to suboptimal living conditions [73–75]. Hence, the observed variation in shell pigmentation would be expected to reflect geographical variation in environmental quality or stability. If so, our results would suggest that Mulroy Bay is the least optimal of the locations we studied, which is to be expected if cultured scallops are reared under very high densities. This is in accordance with Grefsrud *et al.* [72], who showed that cultured scallops seem to be more vulnerable to developmental abnormalities in shell microstructure than wild individuals.

Phenotypic plasticity has long been implicated as a strategy to cope with variable environments, particularly for mollusc species that occupy heterogeneous habitats and have high dispersal potential, including many marine gastropods with planktonic larvae (e.g. [76,77]). Evidence for plasticity (or conversely for the absence of a heritable genetic component) comes from a variety of sources, including reciprocal transplant experiments [78,79] and quantitative genetic studies [80]. However, transplant experiments are not always either possible or desirable from an ecological standpoint, so an increasing number of studies are using genetic markers such as mitochondrial DNA or microsatellites. The premise of these studies is that where phenotypic differences are present among genetically similar populations, it is unlikely that such differences are due to the presence of discrete, locally adapted genetic lineages. In some cases, these genetic studies have shown that morphologically atypical populations are genetically differentiated, indicating a possible correlation between phenotype and genotype [81], whereas other studies have failed to find genetic differences between populations that differ in shell morphology [3,9,82,83].

Although the latter studies are suggestive of phenotypic plasticity, the inability to reject the hypothesis of population differentiation is strictly speaking not proof that there are no genetic differences between populations. We attempted to overcome this issue by increasing the genetic resolution from the 10 or so microsatellites typical of most studies to over 10 000 SNPs and by using a modern morphometric approach capable of providing highly detailed size-independent characterization of shape variability. Having done so, we still found no clear evidence for genetic structure after having excluded Mulroy Bay (electronic supplementary material, figure S7), which lends stronger than usual support to the hypothesis that shell shape and colour have a plastic component. Nevertheless, phenotypic differences need not necessarily reflect genome-wide divergence, because local adaptation can still potentially occur even under high levels of gene flow [84]. Thus, it is possible that phenotypic variation could be associated with one or a small number of loci. These will be very difficult to detect in many natural populations, even with a very large panel of unmapped neutral markers, as linkage disequilibrium tends to decay very rapidly with physical distance in the genome [85–87]. Unfortunately, our sample size of RAD-sequenced individuals was too small to carry out meaningful tests for outlier loci, but our study does support a recent simulation study [88] by showing that as few as five samples per population can be adequate for detecting fine-scale population structure, as suggested also by our power analysis.

## 5. Conclusion

We compared the ability of microsatellites and RAD sequencing to resolve fine-scale population genetic structure in a benthic invertebrate, the great scallop. Our results lend support to previous simulation and empirical studies by showing that even with as few as five individuals per population, RAD sequencing is capable of detecting population genetic structure where a standard panel of microsatellites struggles. Furthermore, a number of populations that exhibited significant phenotypic differences could not be distinguished from one another genetically, suggesting that shell shape and coloration are likely to be phenotypically plastic.

## Supplementary Material

Supplementary table 1: Details of the 15 microsatellite loci used in this study together with their polymorphism characteristics in 180 great scallops sampled from nine different populations

## Supplementary Material

Supplementary table 2: Pairwise Fst values (below diagonal) and corresponding p-values (above diagonal) calculated using (a) 13 microsatellites genotyped in 180 individuals and (b) 10,539 SNPs genotyped in 45 individuals

## Supplementary Material

Supplementary table 3: Pairwise Fst values (below diagonal) and corresponding p-values (above diagonal) calculated using13 microsatellites genotyped in 45 individuals

## Supplementary Material

Supplementary table 4: Summary of the results of the General Linear Mixed Models (GLMMs) with response variables shell shape PC1 and CI, population included as a random effect, and PC1 and PC2 from the PCA of the SNP dataset fitted as a predictor variables

## Supplementary Material

Supplementary table 5: Summary of the results obtained from different de novo assemblies of the ddRAD data generated using different values for three main parameters −m, −M and −n within the denovo_map.pl Script in Stacks. −m and −M define the minimum number of raw reads and the maximum number of mismatches between loci when creating a stack within the same individual respectively. −n corresponds to the number of mismatches allowed between loci when processing multiple individuals. For each tested combination of parameters, we report the total number of tags, the number of tags present in all of the individuals, observed heterozygosity, average depth of coverage, and the number of SNPs obtained after filtering

## Supplementary Material

Supplementary figure 1: Illustration of the process of generating scallop shell outline coordinates for the geometric morphometrics analysis. From left to right: scaled digital photograph, isolated shell outline, and 1000 pseudo-landmarks placed along the shell perimeter

## Supplementary Material

Supplementary figure 2: Mean +/− SE Ln P(D) values of five replicate Structure runs for each value of K, the hypothesised number of clusters in the data, ranging from one to nine, based on (a) 180 individuals genotyped at 13 microsatellites; and (b) 10,539 SNPs genotyped in 45 individuals

## Supplementary Material

Supplementary figure 3: Heat map depicting pairwise Fst values calculated using a restricted dataset of 45 individuals genotyped at 13 microsatellites.

## Supplementary Material

Supplementary figure 4: Results of the Structure analysis of a restricted dataset of 45 individuals genotyped at 13 microsatellites. Panel (a) shows the mean +/− SE Ln P(D) values of five replicate Structure runs for each value of K, the hypothesised number of clusters in the data, ranging from one to nine. Panel (b) shows the resulting cluster membership coefficients of each individual

## Supplementary Material

Supplementary figure 5: Results of shell outline analysis. (a) Contribution of the first three shape variables to the outline description. The shape variability was represented for increasing values along each PC (−3s.d., mean, +3s.d.). (b) Deformation grid depicting the bindings required to pass the average shape for population one to the average shape for population three

## Supplementary Material

Supplementary figure 6: Heat maps depicting pairwise Fst values calculated using SNP datasets derived from different runs of the denovo_map.pl script in Stacks in which different values were used for the three main parameters −m, −M and −n

## Supplementary Material

Supplementary figure 7: Results of the Structure analysis after having excluded samples collected from Mulroy Bay. Results are shown for 13 microsatellites (panels a and b) and 10,539 SNPs (panels c and d). Panels (a) and (c) show mean +/− SE Ln P(D) values of five replicate Structure runs for each value of K, the hypothesised number of clusters in the data, ranging from one to nine. Panels (b) and (d) show the resulting cluster membership coefficients of each individual

## Supplementary Material

Supplementary information 1: Design of the P7 adapters used during the preparation of the ddRAD library

## Supplementary Material

Supplementary information 2: Detailed ddRAD library preparation protocol

## Supplementary Material

Supplementary information 3: Computer code and accompanying documentation for morphometrics and colourimetric analyses

## Data accessibility

Raw reads have been deposited into the Sequence Read Archive (SRA accession: SRP080989). Microsatellite data are available from the Dryad Digital Repository: http://dx.doi.org/10.5061/dryad.mk860 [89].
