# Supplementary figures and images for: RAD sequencing resolves fine-scale population structure in a benthic invertebrate: implications for understanding phenotypic plasticity
Source: R Soc Open Sci. 2017 Feb 8;4(2):160548. doi: 10.1098/rsos.160548 (PMC5367306; doi:10.1098/rsos.160548)

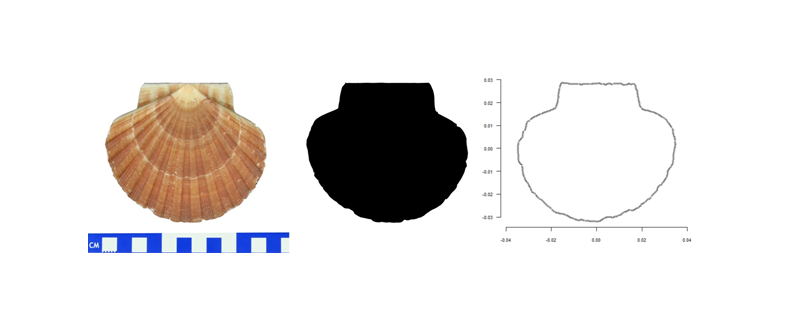

Supplement: Supplementary figure 1: Illustration of the process of generating scallop shell outline coordinates for the geometric morphometrics analysis. From left to right: scaled digital photograph, isolated shell outline, and 1000 pseudo-landmarks placed along the shell perimeter [file rsos160548supp6.tif]

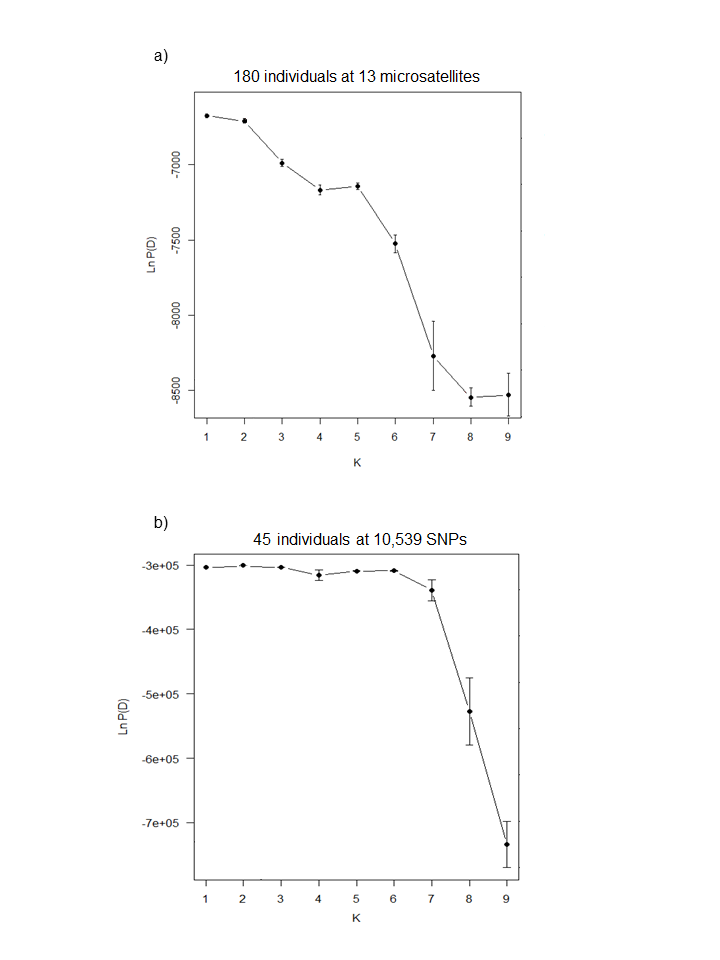

Supplement: Supplementary figure 2: Mean +/− SE Ln P(D) values of five replicate Structure runs for each value of K, the hypothesised number of clusters in the data, ranging from one to nine, based on (a) 180 individuals genotyped at 13 microsatellites; and (b) 10,539 SNPs genotyped in 45 individuals [file rsos160548supp7.tif]

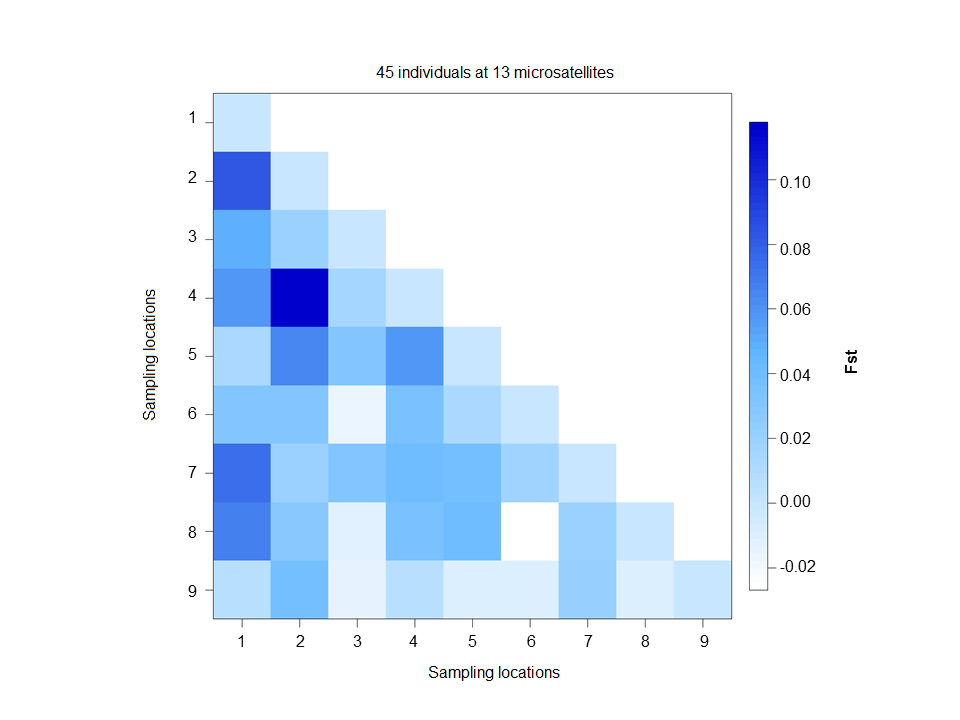

Supplement: Supplementary figure 3: Heat map depicting pairwise Fst values calculated using a restricted dataset of 45 individuals genotyped at 13 microsatellites. [file rsos160548supp8.tif]

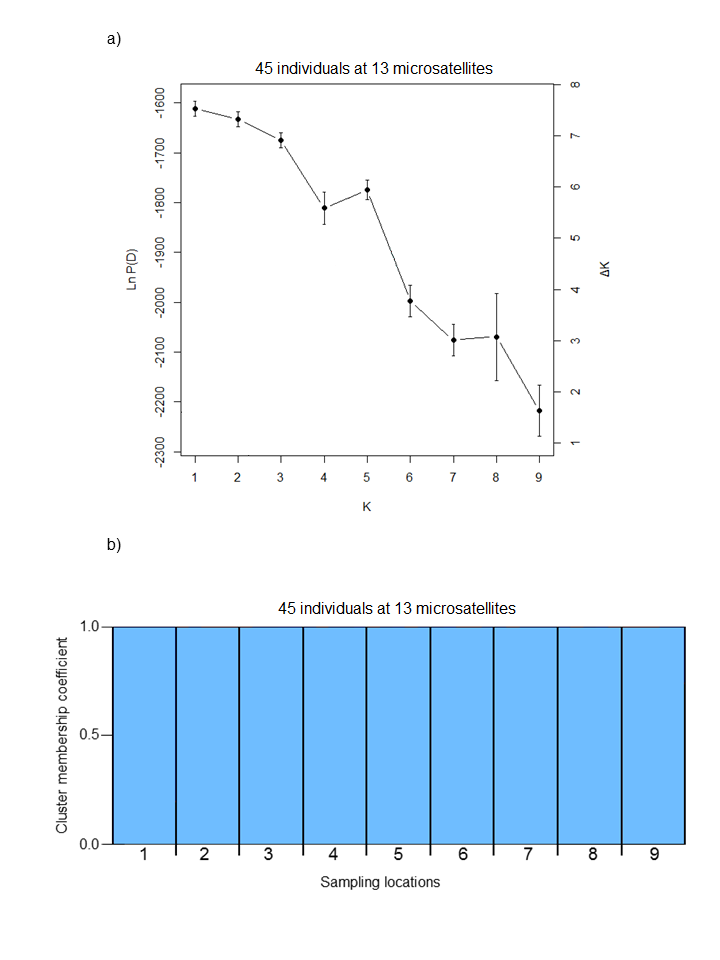

Supplement: Supplementary figure 4: Results of the Structure analysis of a restricted dataset of 45 individuals genotyped at 13 microsatellites. Panel (a) shows the mean +/− SE Ln P(D) values of five replicate Structure runs for each value of K, the hypothesised number of clusters in the data, ranging from one  [file rsos160548supp9.tif]

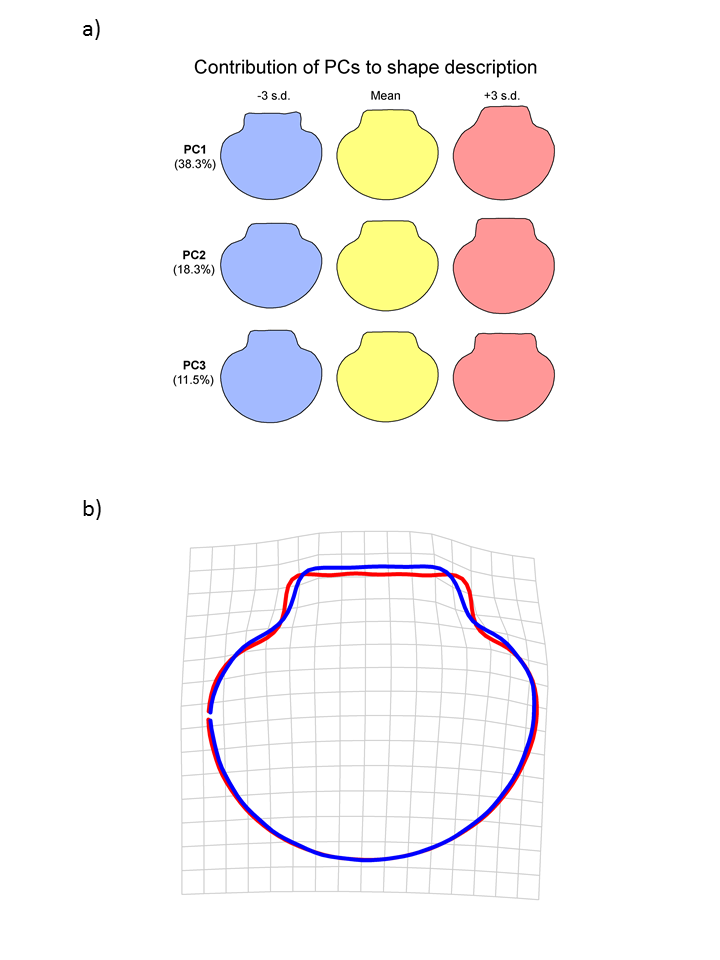

Supplement: Supplementary figure 5: Results of shell outline analysis. (a) Contribution of the first three shape variables to the outline description. The shape variability was represented for increasing values along each PC (−3s.d., mean, +3s.d.). (b) Deformation grid depicting the bindings required to pass th [file rsos160548supp10.tif]

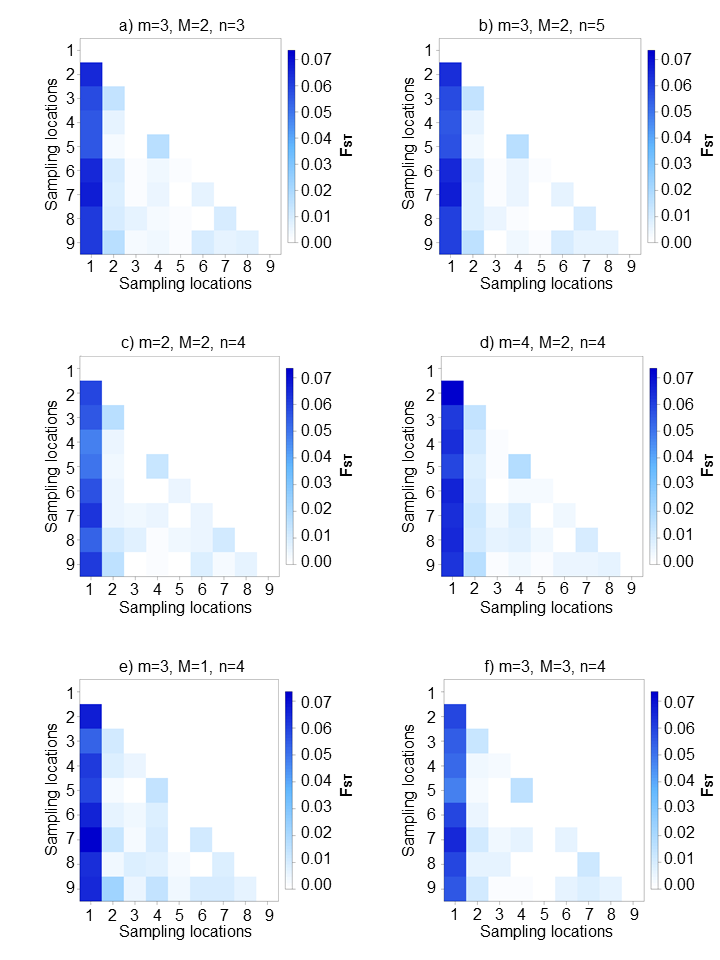

Supplement: Supplementary figure 6: Heat maps depicting pairwise Fst values calculated using SNP datasets derived from different runs of the denovo_map.pl script in Stacks in which different values were used for the three main parameters −m, −M and −n [file rsos160548supp11.tif]

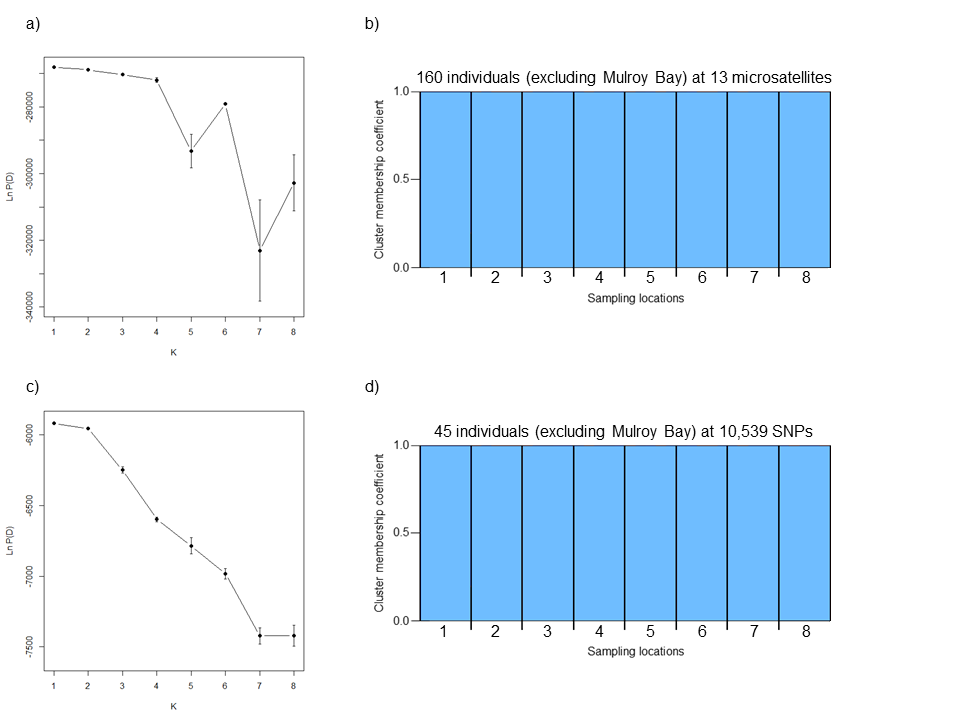

Supplement: Supplementary figure 7: Results of the Structure analysis after having excluded samples collected from Mulroy Bay. Results are shown for 13 microsatellites (panels a and b) and 10,539 SNPs (panels c and d). Panels (a) and (c) show mean +/− SE Ln P(D) values of five replicate Structure runs for each  [file rsos160548supp12.tif]
