# Supplementary material for: RAD sequencing resolves fine-scale population structure in a benthic invertebrate: implications for understanding phenotypic plasticity
Source: R Soc Open Sci. 2017 Feb 8;4(2):160548. doi: 10.1098/rsos.160548 (PMC5367306; doi:10.1098/rsos.160548)
Supplement: Supplementary information 2: Detailed ddRAD library preparation protocol [file rsos160548supp14.docx]

**RNA digestion:**

1. Digest DNA with 1 µl (= 10 µg) RNase A (Thermo Scientific) for 30 minutes at 37°C.
2. Purify with the MinElute Reaction cleanup kit (Qiagen) using manufactures protocol. Elute in 26 µl EB buffer.
3. Measure the DNA concentration with Qubit Fluorometer using the Qubit dsDNA BR assys kit (both Life Technologies) and 1 µl DNA.

**Double digestion:**

1. Use 800 ng of DNA.
2. Double digest setup (Enzymes from Thermo Scientific):

|  | **Vol per 45 µl** |
| --- | --- |
| FD buffer | 4.50 µł |
| NsiI (FastDigest) | 2.25 µl |
| MspI (FastDigest) | 2.25 µl |
| DNA |  |
| Water | Add to 45 µl |

1. Incubate the reaction for 30 min at 37 °C.
2. Cleanup with the MinElute reaction cleanup kit. Eluate in 23 µl sterile water.

**Adapter Ligation:**

1. Apply whole sample volume from the double digestion to the ligation.
2. Ligation setup (Enzymes from New England Biosystems; T4 Ligase 2,000,000 U/ml):

|  | **Vol per 40.5 µl** |
| --- | --- |
| T4 Ligase buffer | 4 µł |
| T4 Ligase | 21 µl |
| P5 Adapter | 5 x * |
| P7 Adapter | 10 x * |
| Sample | 0.5 µl |
| Water | Add to 45 µl |

* Estimate the amount using the Mol Calculator of Peterson et al. (2012) with adapter

excess set of 5 x for P5 and 10 x for P7 relative to the expected number of cut sites.

1. Incubate the reaction for 2 h at 16 °C.
2. Cleanup with the MinElute reaction cleanup kit. Eluate in 52 µl sterile water.

**Size Selection:**

1. 1 meter distance to the magnet:

a) Prepare freshly min. 180 µl 85 % ethanol per sample

b) Shake the SPRIselect bottle to dissolve the beads

c) Add 50 µl sample to 45 µl SPRIselect in PCR-stripes

d) Mix by pipetting 10 x

e) Incubate for 1 min

1. On the magnet:

a) Incubate for 2 min

b) Remove and discard the clear supernatant with a pipette (do not discard the beads)

c) Add 180 µl 85 % Ethanol to the beads on the magnet

d) Incubate for 30 sec

e) Remove and discard the clear supernatant with a pipette (do not discard the beads)

f) Incubate for 5 to 10 min with open lid to remove the rest of the ethanol

1. 1 meter distance to the magnet:

a) Add 28 µl sterile water to each sample

b) Mix by pipetting 10 x

1. On the magnet:

a) Incubate for 1 min

b) Pipette the supernatant to a new tube (includes DNA)

**PCR Amplification:**

1. PCR setup (Q5 polymerase from New England Biosystems):

|  | **Stock** | **Vol per 50.5 µl** |
| --- | --- | --- |
| Q5 buffer | 5 x | 10 µl |
| Q5 Taq | 2,000,000 U/µl | 0.5 µl |
| dNTPs | 2 mmol/l | 5 µl |
| P5 Primer | 10 µmol/l | 5 µl |
| P7 Primer | 10 µmol/l | 5 µl |
| DNA |  | 2.5 µl |
| water |  | 22.5 µł |

1. Temperature profile of the PCR

| Step | Time | Temperature | Cycles |
| --- | --- | --- | --- |
| Initial Denaturation | 30 sec | 98 °C |  |
| Denaturation | 10 sec | 98 °C | 14 cycles |
| Annealing | 30 sec | 65 °C |  |
| Elongation | 30 sec | 72 °C |  |
| Final Elongation | 5 min | 72 °C |  |

1. Run 1.5 % Agarose gel with 5 µl of PCR product to check the PCR success.

**PCR Purificaiton:**

1. 1 meter distance to the magnet:

a) Prepare freshly min. 400 µl 70 % ethanol per sample

b) Shake the Ampure XP bottle to dissolve the beads

c) Add 81 µl Ampure XP to 45 µl PCR product in PCR-stripes

d) Mix by pipetting 10 x

e) Incubate for 1 min

1. On the magnet:

a) Incubate for 2 min

b) Remove and discard the clear supernatant with a pipette (do not discard the beads)

c) Add 200 µl 70 % Ethanol to the beads on the magnet

d) Incubate for 30 sec

e) Remove and discard the clear supernatant with a pipette (do not discard the beads)

f) Add 200 µl 70 % Ethanol to the beads on the magnet

g) Incubate for 30 sec

h) Remove and discard the clear supernatant with a pipette (do not discard the beads)

f) Incubate for 5 to 10 min with open lid to remove the rest of the ethanol

1. 1 meter distance to the magnet:

a) Add 30 µl sterile water to each sample

b) Mix by pipetting 10 x

1. On the magnet:

a) Incubate for 1 min

b) Pipette the supernatant to new PCR stripes (includes DNA)

**Dual Size Selection:**

1. 1 meter distance to the magnet:

a) Prepare freshly min. 180 µl 85 % ethanol per sample

b) Shake the SPRIselect bottle to dissolve the beads

c) Add 28 µl sample to 15.4 µl SPRIselect in PCR-stripes

d) Mix by pipetting 10 x

e) Incubate for 1 min

1. On the magnet:

a) Incubate for 2 min

b) Pipette the supernatant to a new tube (includes DNA)

1. 1 meter distance to the magnet:

a) Add 8.4 µl SPRIselect to the supernatant in the PCR-stripes

b) Incubate for 1 min

1. On the magnet:

a) Incubate for 2 min

b) Remove and discard the clear supernatant with a pipette (do not discard the beads)

c) Add 180 µl 85 % Ethanol to the beads on the magnet

d) Incubate for 30 sec

e) Remove and discard the clear supernatant with a pipette (do not discard the beads)

f) Incubate for 5 to 10 min with open lid to remove the rest of the ethanol

1. 1 meter distance to the magnet:

a) Add 28 µl sterile water to each sample

b) Mix by pipetting 10 x

1. On the magnet:

a) Incubate for 1 min

b) Pipette the supernatant to a new tube (includes DNA)

**Pooling:**

1. Measure the DNA concentration of each sample using the Qubit Fluorometer using the Qubit dsDNA HS assay kit (Life Technologies) and 1 µl DNA.
2. Pool equal amounts of DNA from each sample.
3. Cleanup with MinElute Reaction cleanup kit. Eluate in sterile water.

=> Amount needed for the LabChip channels: (x channels * 10 µl water) + 1 µl water

**Final Size Selection:**

1. For at least one channel per lane use the LabChip XTe run the DNA 750 assay kit with a size range of 308 – 462 bp and the extract and stop method at least one channel per lane (max. 1 µg DNA per lane)
2. Cleanup with MinElute Reaction cleanup kit. Eluate in sterile 11 µl water.
3. Measure the DNA concentration of each sample using the Qubit Fluorometer using the Qubit dsDNA HS assay kit (Life Technologies) and 1 µl DNA
