# Supplementary material for: RAD sequencing resolves fine-scale population structure in a benthic invertebrate: implications for understanding phenotypic plasticity
Source: R Soc Open Sci. 2017 Feb 8;4(2):160548. doi: 10.1098/rsos.160548 (PMC5367306; doi:10.1098/rsos.160548)
Supplement: Supplementary information 3: Computer code and accompanying documentation for morphometrics and colourimetric analyses [file rsos160548supp15.pdf]

Supplementary Document 3: Elliptic Fourier analysis of outlines and Colorimetric analysis

R-Markdown Report - R Script Commentary

Contents

1 Elliptic Fourier analysis: outline extraction **2**

2 Collection of coordinates (`Coo` objects) **2**

2.1 Shape graphics: outlines inspection 3

2.2 Outlines adjustement 4

2.3 Normalization 4

3 Outline Analysis (`Coe` objects) **6**

3.1 Calibration 6

3.2 Elliptic Fourier Transformation (`Coe`) 9

4 Multivariate analysis **11**

4.1 Principal component analysis (PCA) 11

4.2 Multivariate analysis of variance (MANOVA) 15

4.3 Conclusions (Multivariate Analysis) 16

5 Mean shape analysis **17**

5.1 Thin plate splines analysis (TPS) 18

6 Colour anlysis: data description **19**

7 Descriptive statistics **19**

7.1 Data normality and homoscedasticity 21

8 Colour Index **22**

8.1 One-way ANOVA 23

9 Colour intensity and area **25**

9.1 Colour intensity analysis 25

9.2 Colour area data analysis 27

10 Conclusions (Colour analysis) **30**

Elliptic Fourier analysis of outlines and colorimetric analysis of *Pecten maximus* from nine populations collected along the Northern Ireland coastline are documented.

1 Elliptic Fourier analysis: outline extraction

The input data for the morphometric analysis of outlines is a set of (x;y) pixel coordinates sampled on each of the image outline. The images preparation can be summarised in the following steps:

1. Photographs of the lateral view of flat (left) valves for each individual were obtained with an high-resolution digital camera (Nikon D3300);
2. Images were prepared with an image software (Adobe Photoshop CS6) in order to get *grey-level* masks (depicting the outlines of the shells) *8-bit* mode in *.jpg* formats with no level of compression;
3. The images were centred so that all the masks were aligned to the same point and stored in the same folder;
4. Only the outlines of intact scallops shells were retained for following analysis (10 outlines out of 180 were excluded).

To summarise, for each shell photo a black mask on a white background was obtained for each shape to be analysed .

```
# Loading the Momocs package
library(Momocs)
library(ggplot2)

# List of .jpg for the outline extraction (just an example)
head(scallop.list, 6) # names of the first six files for site A
```

```
## [1] "1_0.1.jpg" "1_0.2.jpg" "1_0.3.jpg" "1_0.4.jpg" "1_0.5.jpg" "1_0.6.jpg"
```

Each outline was imported directly from the black mask with an algorithm extracting the image outline. In the outline analysis each outline was described through the closed polygon formed by the (x;y) coordinates of the pixels defining it.

```
# Outlines extraction and creation of a list of (x;y) coordinates
scallop.out <- import_jpg(scallop.list)
```

In order to specify explanatory variables going along with the extracted outline coordinates, **grouping factors** and **covariates** were retrieved from the image file-names. These factors were specified through a data-frame, and then used to create subsets in the collection of coordinates (see below the definition of `Coo` objects).

```
# Retriving covariables from the file-names and defining the grouping factors
grouping.fac <- lf_structure(scallop.list, names = c("Site", "Number"),
                             split = "_", trim.extension = T)
```

2 Collection of coordinates (`Coo` objects)

After the definition of a list of coordinates for each of the shell image, the list was passed to one of the Momocs class builder, "`Out`"" (class builder for closed outlines) and a `Coo` (Collection of COOrdinates) class object defined, along with grouping factors (Site).

```
## # Definition of a Coo and grouping factors
## scallop.coo <- Out(scallop.out, fac = grouping.fac[1])
```

```
## An Out object with:
## --------------------
##  - $coo: 170 outlines (4642 +/- 461 coordinates, all closed)
##  - $fac: 1 classifier:
##      'Site' (factor 9): 1, 2, 3, 4, 5, 6, 7, 8, 9.
```

Before outline analysis, shapes were organised into a collection of coordinates, a `Coo` object. The `Coo` object carries:

- a component names `$coo`, a list of 170 **shapes** with the outline type (closed) and average number of points sampled +/- deviation;
- a component `$fac`, a list of **factors** for classification (Site).

2.1 Shape graphics: outlines inspection

The extracted outlines were investigated with different graphical tools. Single shapes from the collection were investigated as well as the whole `Coo` objects through panel representations.

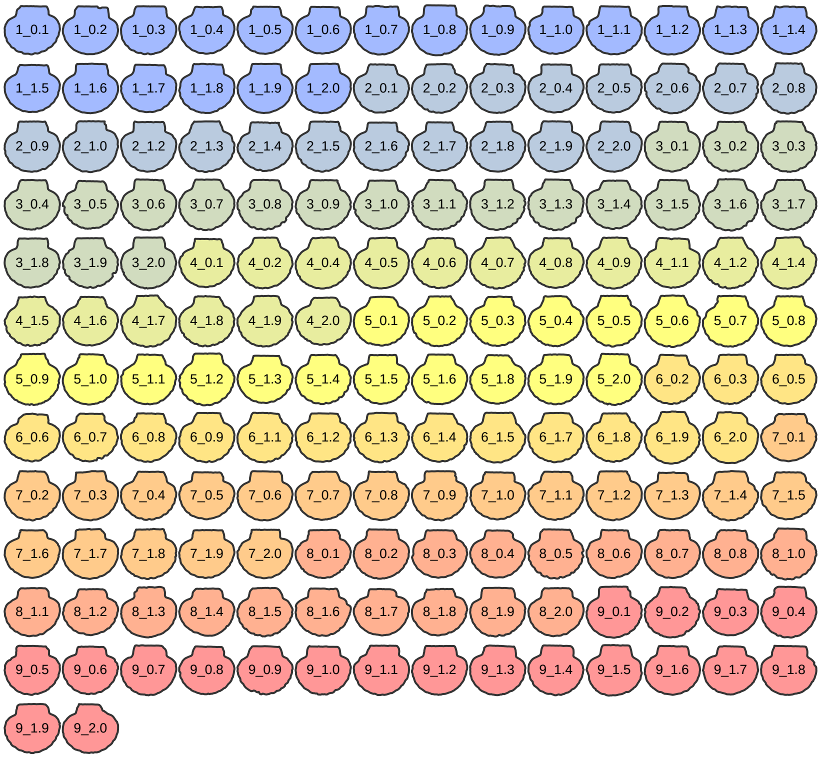

Figure 1: Panel with all the extracted outlines from the `Coo`. Individuals with colours matching the different grouping factors (from 1 to 9).

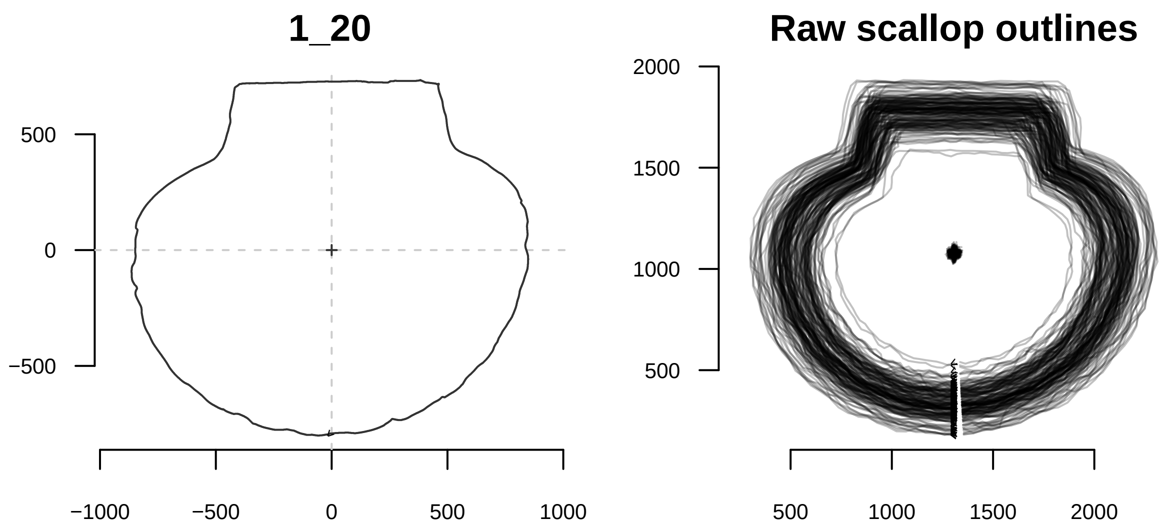

Figure 2: Plot of a single centred random outline (1__20) and all the shapes plotted in the same windows (“+” = shape centroid, “<” = first point).

From the plot it was clear that the outlines were neither centred nor aligned. Also, the first point of the outlines (black arrows) varied along the bottom side (not homologous).
The outlines showed an overall difference in size, rotation and translation.

2.2 Outlines adjustement

On `Coo` object several families of generic operations can be performed:

- plotting functions (shape graphics, see above)
- **geometric operations** (e.g. alignments, centring, rescaling etc.)

Usually the outlines become rough due to artefacts during the manual digitization processes, therefore a specific number of **smoothing** iterations was selected.

```
# Outlines smoothing (5 smoothing iterations)
scallop.coo <- coo_smooth(scallop.coo, 5)
```

2.3 Normalization

The concept of **normalization** in Elliptical Fourier analysis is central and has long been a matter of trouble. There are two ways of normalizing outlines. The first, and by far the most used, is to use a “numerical” alignment directly on the matrix of coefficients. The coefficients of the **first harmonic** are consumed by this process but higher-rank harmonics are normalized for size and rotation. This is sometimes referred as using the “first ellipse”, as the first harmonic defines the best-fitting ellipse in the plane that is consumed during the normalization.

The problem with this method is that biases can be introduced if your shapes are prone to bad alignments among all the first ellipses. This is usually easy to observe on the morphospace where upside-down shapes (or rotated), bizarre clustering and outline deformations can be detected. Most of the time this is due to a poor normalization on the matrix of coefficients, and the variability observed is caused by the variability in the

alignment of the first ellipse used for the normalization. The shapes prone to this are usually rounded/ellipsoid with a strong bilateral symmetry.

When this happen, another method of normalization should be used and shapes are aligned through **geometric operations** before the Elliptic Fourier transformation and the latter is performed with no normalization. With this method shapes are aligned before the transformation with operations performed directly on the coordinates or on some landmarks along the outline, such as **Procrustes alignment** on pseudo-landmark. First point should be made homologous as well to minimize any subsequent problems.

In this case, since the shapes were prone to bad alignments among all the first ellipses, the normalization of outlines was carried before the Elliptic Fourier Transformation through the following steps:

1. The outlines were first **centred** (so all the centroid were moved to the same coordinates) and **rescaled**;
2. **Procrustes superimposition** was used to obtain a optimal alignment of the outlines with the same numbers of sampled coordinates (1000 pseudo-landmarks);
3. The **starting point** was also normalized (made homologous) for the following analysis to minimize any subsequent bias.

To compare the shapes of two or more objects, they must be first optimally aligned or "superimposed". **Procrustes superimpositions** (PS) aims to minimize the sum of squared distances between similar landmarks or fixed coordinates by allowing size, rotation and translation to be adjusted.

```
# Centering outlines
scallop.coo.adj  <- coo_center(scallop.coo)
# Re-scaling and point sampling (1000 points)
scallop.coo.adj  <- coo_scale(scallop.coo.adj)
scallop.coo.adj <- coo_sample(scallop.coo.adj, 1000)
# Procrustes superimposition
scallop.coo.adj <- fgProcrustes(scallop.coo.adj)
# Normalization of the starting point
scallop.coo.adj <- coo_slidedirection(scallop.coo.adj, "W")
```

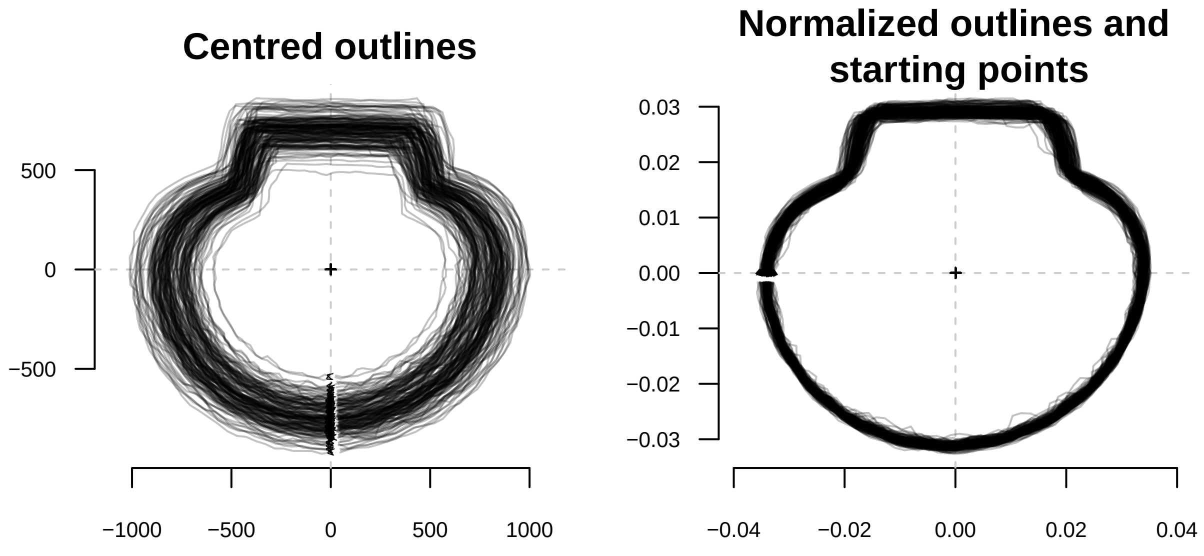

Figure 3: Centred shapes and Outline resulting from Procrustes superimposition

3 Outline Analysis (`Coe` objects)

3.0.1 Elliptic Fourier analysis

As described, the method fits separately the X and Y coordinates of an outline projected on a plane. This approach is very popular since it has great advantages: equally spaced points are not required and the coefficient can be made intendant of outline position and normalized for size. Four coefficients per harmonic are obtained with this method and normalisation of coefficients can be performed. They can be also normalised for the location of the first outline coordinates, so that shapes are individually aligned according to their first-fitted ellipse.

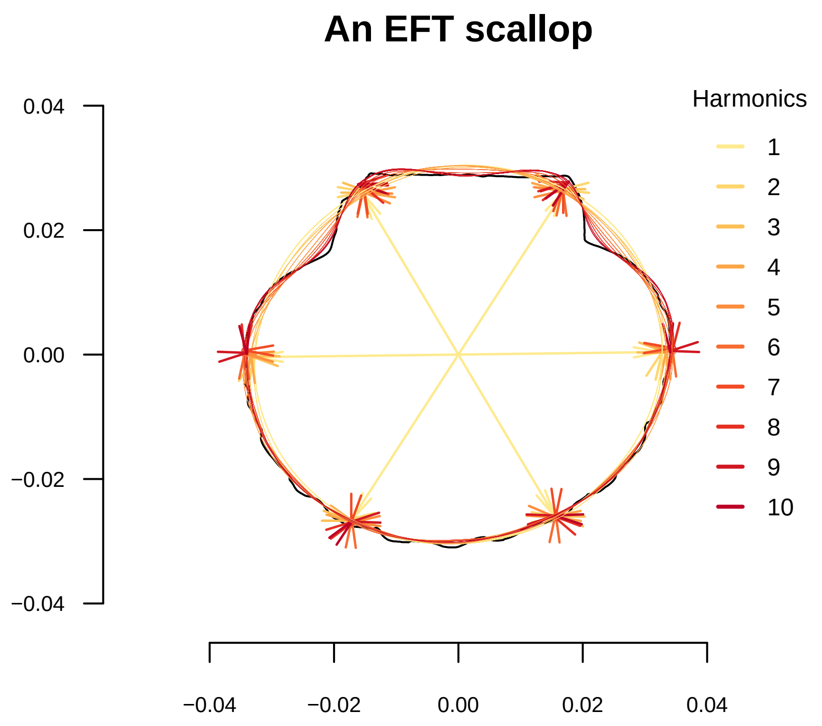

Figure 4: Ptolemaic ellipses which illustrate intuitively the principle behind elliptical Fourier analysis.

3.1 Calibration

The advantage of the Fourier-based approaches is that they can fit virtually any shapes, while the ratio signal/noise can be quite small for higher levels harmonics. This means that the details described by the high frequency harmonics are due to many things, e.g. digitalization error, and not to real difference among shapes.
Therefore, a critical question in outline Fourier analysis is: *what is the right number of harmonics?* So far, there is no objective criterion, since it depends uniquely of the scope of the study and the level of details we want from the analysis. But there are some approaches that can be used to assess the most appropriate number of harmonics before carrying out a Fourier transformation (e.i. shape recostruction, deviation and harmoic power analysis).

3.1.1 Shape reconstruction

This method allowed a first qualitative estimation of the number of harmonics to be used. The approach consists to observe the reconstructed mean shape for a given range of harmonics. The number of harmonics providing a satisfactionary, or almost perfect, recostruction of the outline can be selected.

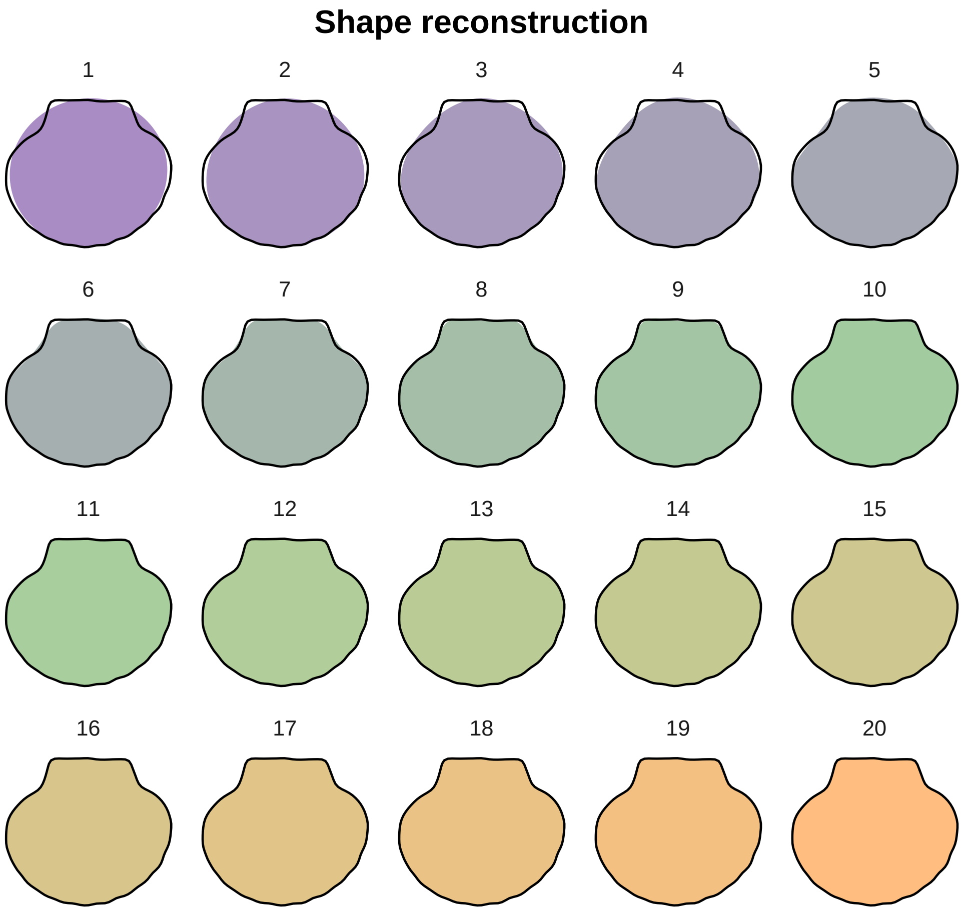

Figure 5: Mean shape reconstructed (coloured backgrounds) for an increasing number of selected harmonics. Thirteen - eighteen harmonics provided an almost perfect recostruction of scallop outlines.

3.1.2 Deviation

The idea of deviation approach was to define the best possible fit for a given number of sampled points along the outline and then calculated the deviation, in terms of euclidean distance, between fits with a different range of harmonics and the best fit for each of the sampled point.

The deviation was then normalized by the centroid size and represented as "% of the centroid size". The most appropiate number of harmonics to select could be the one leading to an avarage deviation of 0.005.

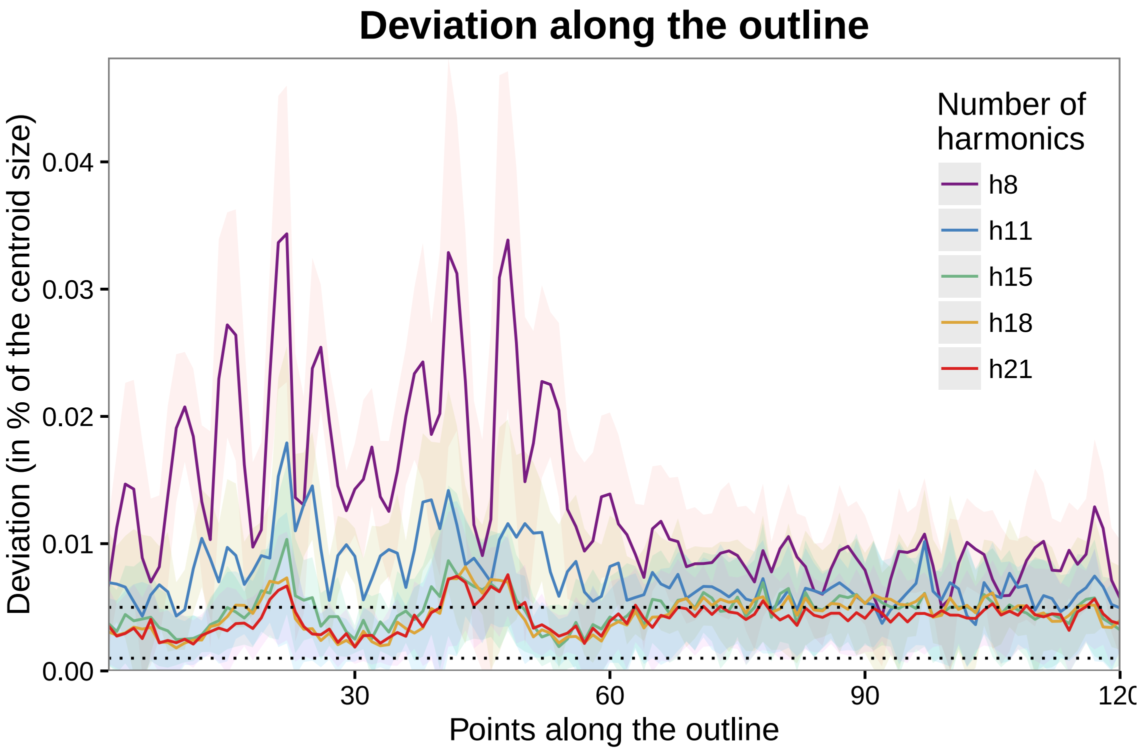

Figure 6: Deviation between the best possible fit and a given range of harmonics (8, 10, 15, 18, 21) for each point (continuous line) and standard error (backgroud); y-axis represents the deviation as *% of the centroid size* for every sampled point (x-axis). Dotted lines represent avarage deviation of 0.001 and 0.005 respectively.

For a given number of sampled points along the outline, a sufficiently small avarage deviation (0.005) from the best fit was provided by 15 harmonics.

3.1.3 Harmonic power

The number of harmonics was also estimated after examining the spectrum of harmonic Fourier power. The power is considered as a measure of the shape information explained by an harmonic function As the rank of the harmonic increases the power decreases adding less and less information. The power for a give harmonic is calculated:

$$Power_n = \frac{A_n^2 + B_n^2 + C_n^2 + D_n^2}{2}$$

where $A_n,\ B_n,\ C_n,\ D_n$ are the harmonic coefficients for the n harmonic.

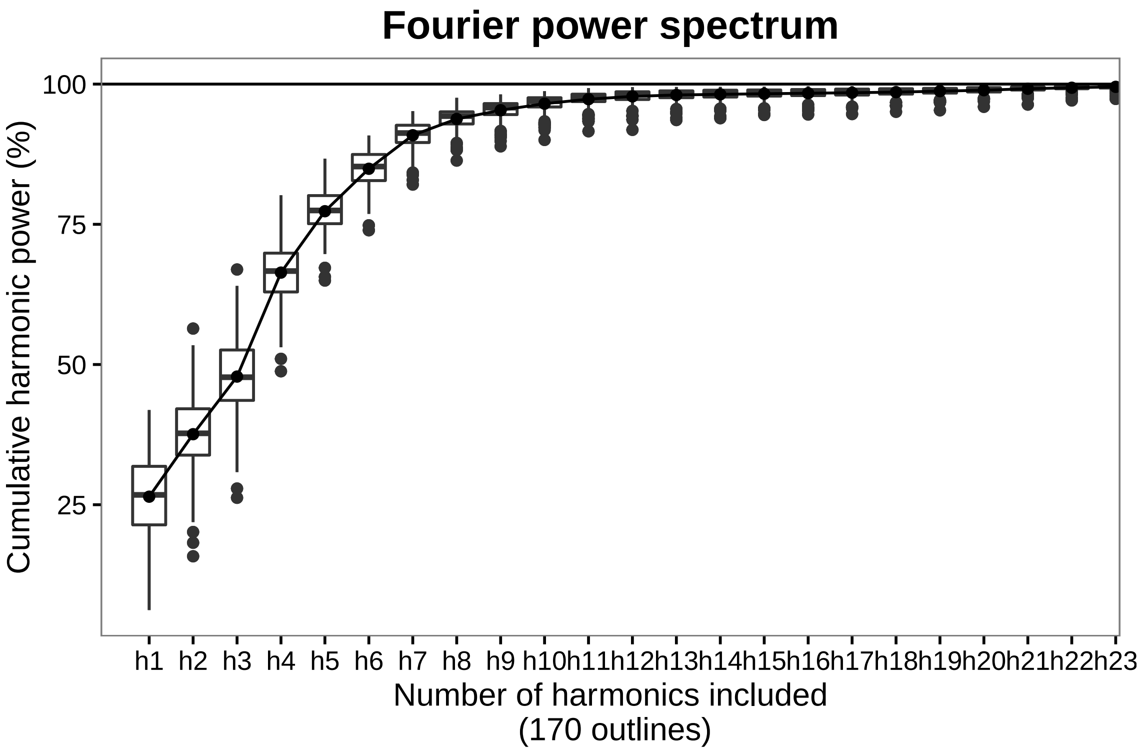


```
##   90%   95%   99% 99.9%
##     8    10    21    30
```

Figure 7: Cumulative harmonic Fourier power for the scallop outlines.

Twenty-one harmonics gathered the 99% of the total harmonic power. Therefore they gathered the 99% of the "shape information", while the remaining 1% should be considered the contribution of harmonics describing outlines details mainly due to digitization noise.

How many harmonics?

A comparison of the above methods showed that 21 harmonics effectively captured 99% of the total harmonic power, but the effective number of harmonics providing a reliable shape recostruction and an acceptable deviation is significantly lower (between 13 and 18 harmonics).

Therefore, 15 harmonics were selected as an optimal compromise in order to provide a reliable shape recostruction by capturing a sufficiently high proportion of shape information (98% of harmonic power) and to enhance robustness of statistical analysis (PCA) by reducing significantly the number of descriptors (harmonic coefficients) calculated for each individual (from 84 - 21 harmonics to 60 - 15 harmonics).

3.2 Elliptic Fourier Transformation (`Coe`)

Once the right number of harmonics was determined an Elliptic Fourier transformation was performed on the list of coordinates (`Coo`) in order to extract the geometric information contained in the outlines. The transformation was carried out without normalization, since it had already been performed directly on outlines through geometric operations. When a morphometric method is applied to the `Coo` object, it is turned into a matrix of harmonic coefficients. Specifically, for each outline "4 $\times$ *Number of Harmonics*" coefficients were obtained.

```r
# Elliptic Fourier analysis (EFT)
scallop.coe <- efourier(scallop.coo.adj, nb.h = 15, norm = FALSE) # without normalization
scallop.coe
```

```
## An OutCoe object [ elliptical Fourier analysis ]
## --------------------
##  - $coe: 170 outlines described, 15 harmonics
##  - $fac: 1 classifier:
##      'Site' (factor 9): 1, 2, 3, 4, 5, 6, 7, 8, 9.
```

Morphometrics on coordinates produced a `Coe` object (Collection of COEfficients). The `Coe` object carried similarly to a Coo:
* a component names `$coe`, a matrix of 4 coefficients extracted from 15 harmonics for each of the 170 outlines;
* a component `$fac`, a list of **factors** for classification (Site).

In this way, the geometric information contained in the outlines were quantified. Since all the coefficients could be considered as quantitative variables, measuring the shell shape variability, generic and specific operations could be applied to the `Coe` object, as well as statistical methods.
Before multivariate analysis was performed, the results of the elliptic Fourier analysis were explored in order to check: *i*) the **harmonic contribution** to the shape reconstruction and *ii*) the **coefficients variability** along the whole dataset.

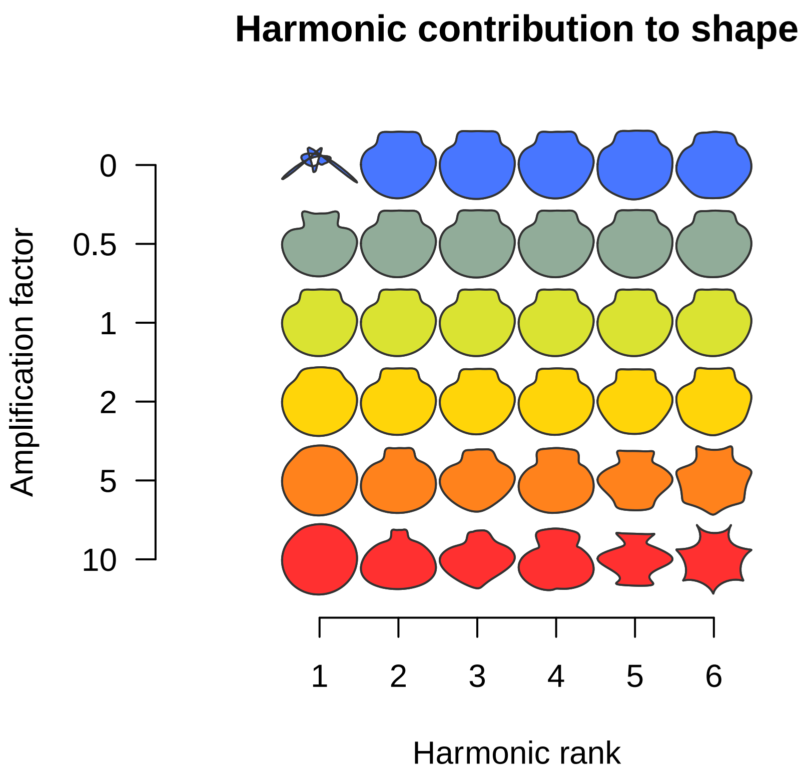

Figure 8: Harmonic contribution of every harmonic to the shape reconstruction.

The graph showed the contribution of every harmonics (first 6) to the mean shape. The effect of each harmonic on the mean shape reconstruction was observed when the corresponding coefficients were multiplied

by amplification factors showing their removal (0), the normal shape they lead to (1), and exaggerated effect on shape reconstruction (2, 5, 10 times).
While analysis of **exaggerated coefficients** were very useful to understand the shape contribution of each harmonic:

- 1st *harmonic* contributed to describe the whole roundness of the shell;
- 2nd *harmonic* described the variability in the length of the **hinge area**;
- 3rd *harmonic* contributed to the shape (point/rounded) of the shell **dorso-ventral extremities** (ventral side and hinge area);
- 4th *harmonic* described the length (or angle) of the **contact between the squared hinge area and the rounder shell**;
- $5th and higher order *harmonics* showed a broad effect on the whole shell with no evident effects on identifiable shell parts.

4 Multivariate analysis

Principal component analysis and other multivariate approaches were directly performed on `Coe` object (harmonic coefficients considered as quantitative variables of the shape variability). Two approaches were used:

1. **Principal Component Analysis** (**PCA**) to analyse how the shape variability varied with the quantitative variables;
2. **Multivariate Analysis of Variance** (**MANOVA**) to check for significant differences in shape among populations.

4.1 Principal component analysis (PCA)

A **Principal Component Analysis** using a Singular Value Decomposition method was carried out on harmonic coefficients in order to see if the morphological variability of the scallop shells could be used to explain differences and similarities among the nine scallop populations ($sites = 9, n = 170$). For each of the individual, the number of quantitative variables used was equal to "$4\ Coefficients\ \times\ Harmonics\ n°\ \times\ Outlines$". Specifically, a PCA was carried out to observe how the shape variability (**morphospace**) was spread across the quantitative variables (linear combination of harmonic coefficients or PCs), and where the different population fall on the factorial plane.

```
# Principal Component Analysis (PCA)
scallop.pca <- PCA(scallop.coe)
scallop.pca
```

```
## A PCA object
## --------------------
##  - 170 shapes
##  - $method: [ efourier analysis ]
##  - $fac: 1 classifier:
##      'Site' (factor 9): 1, 2, 3, 4, 5, 6, 7, 8, 9.
##  - All components: sdev, rotation, center, scale, x, fac, mshape, method, cuts.
```

After the eigenvectors were determined and the principal components calculates (PCs=60) the percentage of the total **variance** captured by each PC, as well as the cumulative proportions of variance explained were analysed.

```r
# Percentage variation explained by each PC
summary(scallop.pca)  # first five PCs
```

```
## Importance of components:
##                              PC1       PC2       PC3       PC4       PC5
## Standard deviation     0.0007478 0.0005172 0.0004096 0.0003522 0.0002748
## Proportion of Variance 0.3829200 0.1832000 0.1149200 0.0849200 0.0517100
## Cumulative Proportion  0.3829200 0.5661200 0.6810400 0.7659600 0.8176600
```

The first two PCs explained the 56.6%, while the first three PCs the 68.1% of the variation among sites. PC1, PC2 and PC3 provided a reasonably realistic representation of the differences among samples.

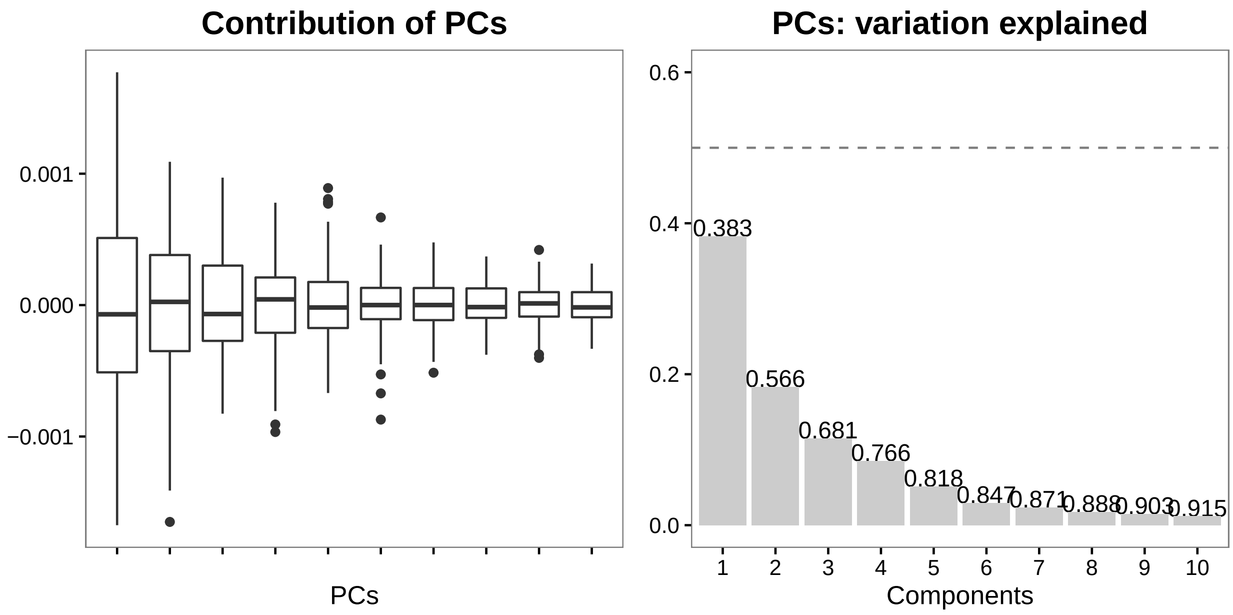

Figure 9: Contribution to description of shape variability and the scree-plot showing the percentage variance explained by the first 10 principal components.

Only the first three principal components (**PC1, PC2, PC3**) were selected to be included in further analysis because they captured a significant amount (68.1%) of the shape variability.

The Principal Components **contribution to the shape description** was also represented as the shape reconstructed for increasing and decreasing values along the PCs ($meanvalue$, ± 2 $s.d.$, ± 3 $s.d$).

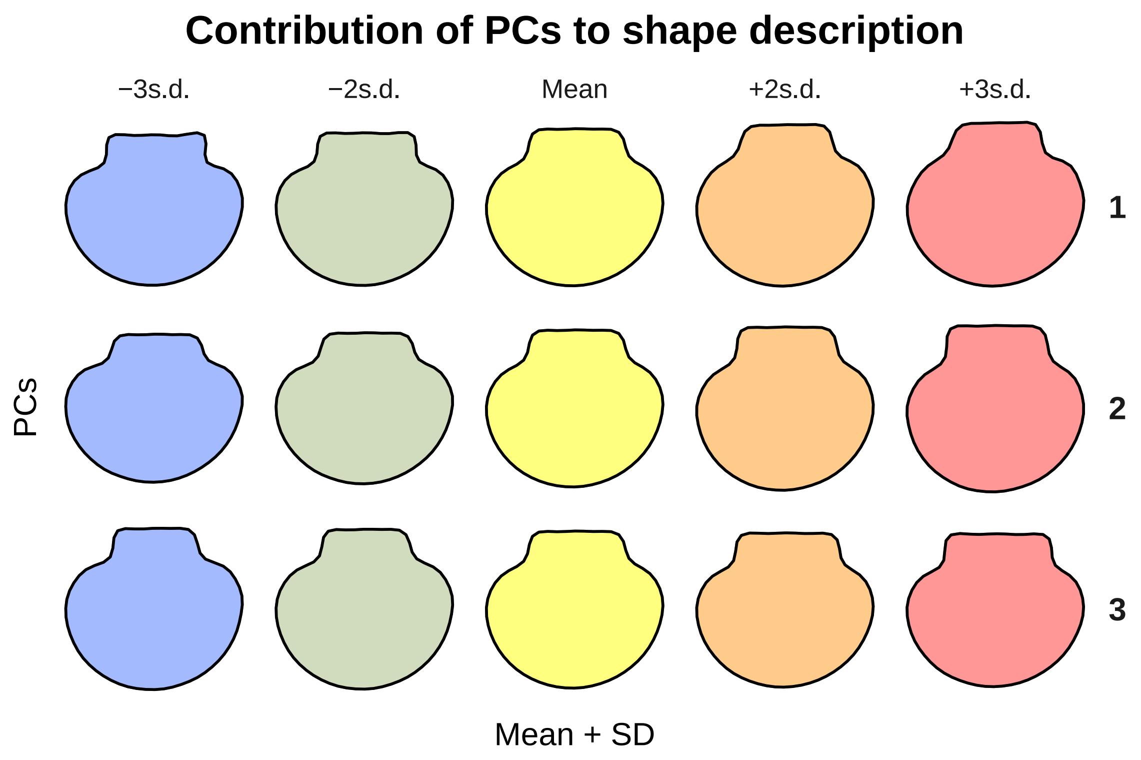

Figure 10: Graph showing the contribution of the PCs to the shape reconstruction for a given position along the PC ($meanvalue$, ± 2 *s.d.*, ± 3 *s.d*).

The PCs (linear combination of harmonic coefficient) showed a different contribution to specific part of the outline:

- the **PC1** highly contributed (38.3%) to the variability in the **"shape"** of the hinge area (variation of the angle formed from the round shell outline and the hinge area): low value corresponded to more "squared"" hinge area, while high values showed a more "pointy" hinge;
- the **PC2** contributed (18.3%) to the variability of the **height** of the hinge; low value showed a short hinge with a more compact shell, while high values showed a well developed hinge with a more elongated shell;
- the **PC3** contributed (11.5%) to the variability of the hinge **length**, small value showed a short hinge, while high values the reverse.

All the PCs showed a marked contribution to the shape variability of the **hinge shape** (angle, height, length). Therefore the **hinge area** was selected as the shell "feature" describing the most of the variability among the nine scallop populations.

The outline variability among the *Pecten maximus* populations was explored through **two-variables scatterplots** of the PCs showing the **morphospace** variability with different contribution of PC1-PC2, PC1-PC3 and PC2-PC3.

The **PC1-2-3** captured the most of the shape variability and were shown to provide a quite consistent group distribution on the **morphospace**.

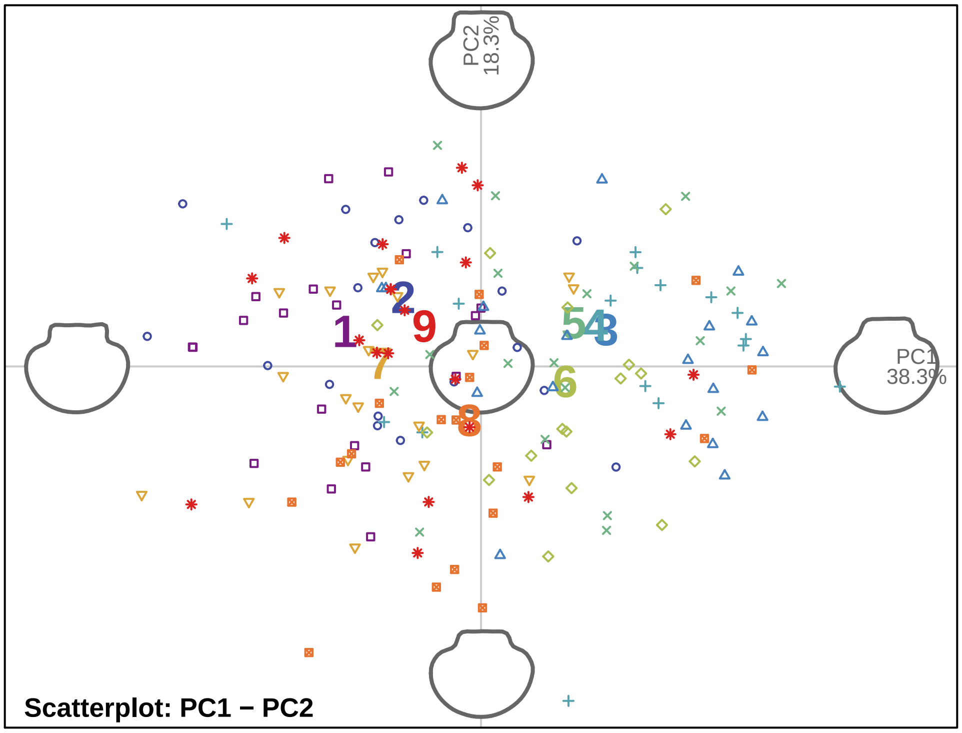

Figure 11: Scatter-plots of PC1-2 showing the spread of groups across the morphospace.

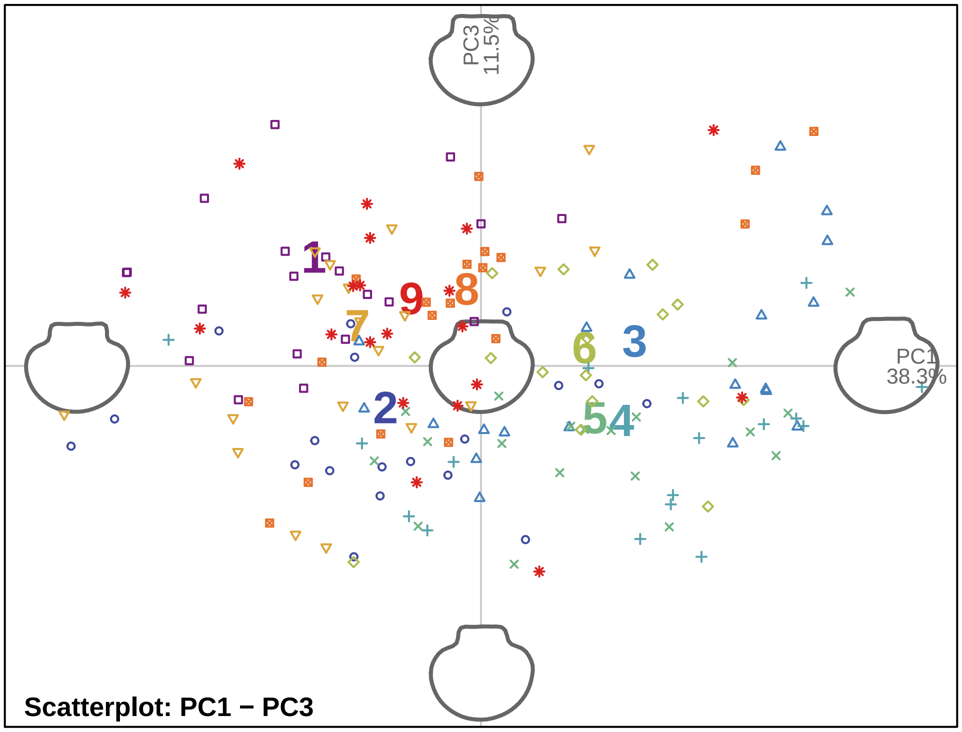

Figure 12: Scatter-plots of PC1-3 showing the spread of groups across the morphospace.

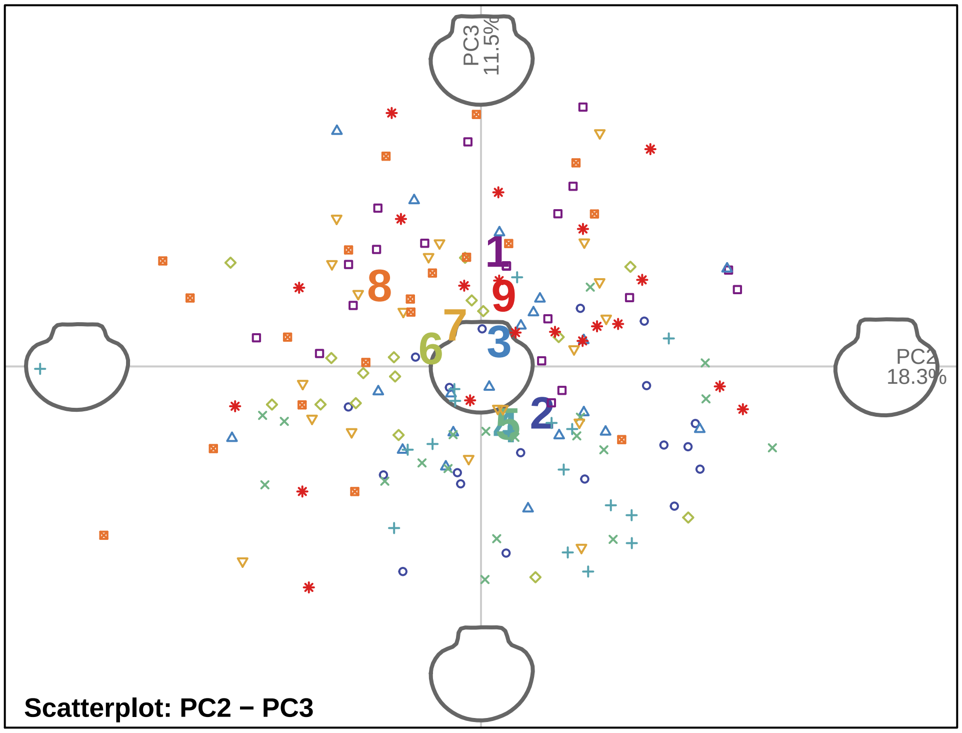

Figure 13: Scatter-plots of PC2-3 showing the spread of groups across the morphospace.

The three scatter-plots showed the shape variability of the nine populations across the morphospace. Along the PC1 (38.3%) the scallop populations were clearly separated, forming distinct clusters on the morphospace. Specifically, the PC1 separated the populations **1, 2, 7, 9** from **3, 4, 5, 6**, while **8** showed intermediate shell shapes. The groups A and A7-8-9 were the ones at the extremes of the morphospace along the PC1.
PC1 was correlated with the **"shape"** of the hinge area (negative correlation with the angle formed from the rounder shell outline and the hinge area); PC2 was positively correlated with the **hinge height**; while PC3 was positively correlated with the **hinge length**.

The most important PCs were extracted and used for the following statistical analysis as a measure of the *"morphological variability"* of individuals.

```
# Extracting the principal components (PC1,PC2,PC3)
scallop.pc1 <- scallop.pca$x[,1]
scallop.pc2 <- scallop.pca$x[,2]
scallop.pc3 <- scallop.pca$x[,3]
scallop.df <- cbind(grouping.fac, scallop.pc1, scallop.pc2, scallop.pc3)
scallop.df <- scallop.df[-2]
```

4.2 Multivariate analysis of variance (MANOVA)

Hypothesis *"There is a significant difference of the pricipal components (shape variability) among different populations"*

The principal components measuring the morphological variability among sites, were analysed with a **Multivariate Analysis of Variance** (**MANOVA**) in order to test if there was a significant shape difference among the nine populations

A multivariate analysis of variance was carried out on the first 15 principal component explaining a cumulative percentage variation of 95%.

```r
# MANOVA
scallop.manova <- MANOVA(scallop.pca, "Site", test = "Wilks", retain = 0.95)
scallop.manova
```

```
##             Df   Wilks approx F num Df den Df    Pr(>F)
## fac          8 0.11196    3.177    120 1058.8 < 2.2e-16 ***
## Residuals 161
## ---
## Signif. codes:  0 '***' 0.001 '**' 0.01 '*' 0.05 '.' 0.1 ' ' 1
```

A **MANOVA** showed a significant difference in shell shape among the 9 scallop populations ($Wilks' \ \lambda \ = 0.112, \ approx - F_{8,161} = 3.18, \ p < 0.0001$).

```r
# MANOVA (without population 1)
scallop.pca.b <- filter(scallop.pca, Site != "1")
scallop.manova.b <- MANOVA(scallop.pca.b, "Site", test = "Wilks", retain = 0.95)
scallop.manova.b
```

```
##             Df   Wilks approx F num Df den Df    Pr(>F)
## fac          7 0.13577    2.889    105 828.61 < 2.2e-16 ***
## Residuals 142
## ---
## Signif. codes:  0 '***' 0.001 '**' 0.01 '*' 0.05 '.' 0.1 ' ' 1
```

A **MANOVA** showed a significant difference in shell shape among the 8 scallop populations ($Wilks' \ \lambda \ = 0.136, \ approx - F_{7,142} = 2.89, \ p < 0.0001$).

4.3 Conclusions (Multivariate Analysis)

A **Principal Component Analysis** showed that the first three principal components accounted for the 68.1% of the variation among the 9 scallop populations. A plot of the PC1-2 and PC1-3 showed a clear separation of the sites **1, 2, 7, 9** from **4, 5, 6, 7**,while **8** showed intermediate shell shapes.
The PC1 (38.3%) was positively correlated with the **"shape"** of the hinge area (variation of the angle formed from the rounder shell outline and the hinge area), PC2 (18.3%) was positively correlated with the **hinge height**, while PC3 (11.5%) showed a positive correlation with the **hinge length**. Because of the percentage variations explained, these three variables were considered the ones that contributed the most to the observed shape differences among samples.

The PC1 - PC2 scatter-plot showed a separation in the morphospace of **1, 2, 3, 7, 9** with more "squared" hinge areas compared to **4, 5**, while **8** showed average hinge shapes. The hinge "shape" was the variable with the strongest contribution to the PC1. This parameter was the one explaining the highest level of variability among the scallop populations.
The PC1 - PC3 showed again a consistent separation of the 9 populations on the morphospace (1, 2, 3, 7, 8, 9 and 4, 5), with the population 1 ant 3, 4, 5 at the extreme of the morphospace.

All the PCs showed a marked contribution to the shape variability of the **hinge area** (angle, height, length). Therefore the **hinge shape** (PC1) was selected as the shell parameters with the strongest contribution to the differences, among the nine scallop populations: 1, 2, 7, 9 with a more "squared" hinge area, and 4, 5 with a more "pointy" hinge.

The morphological differences (expressed through the first 10 PCs) were also tested to be significant among groups. A **MANOVA** showed a significant difference in shell shape (first 10 PCs) among the 9 scallop populations ($Wilks' \ \lambda \ = 0.112, \ approx - F_{8,161} = 3.18, \ p < 0.0001$), even excluding the population 1 ($Wilks' \ \lambda \ = 0.136, \ approx - F_{7,142} = 2.89, \ p < 0.0001$).

5 Mean shape analysis

The mean shell shapes for all the scallops (global) and group wise were retrieved directly from the `Coe` object. These were analysed after superimposition in order to describe differences between the shell outlines at the extreme of the morphospace.

```
# Group wise mean shapes
scallop.mean <- mshapes(scallop.coe, "Site")
```

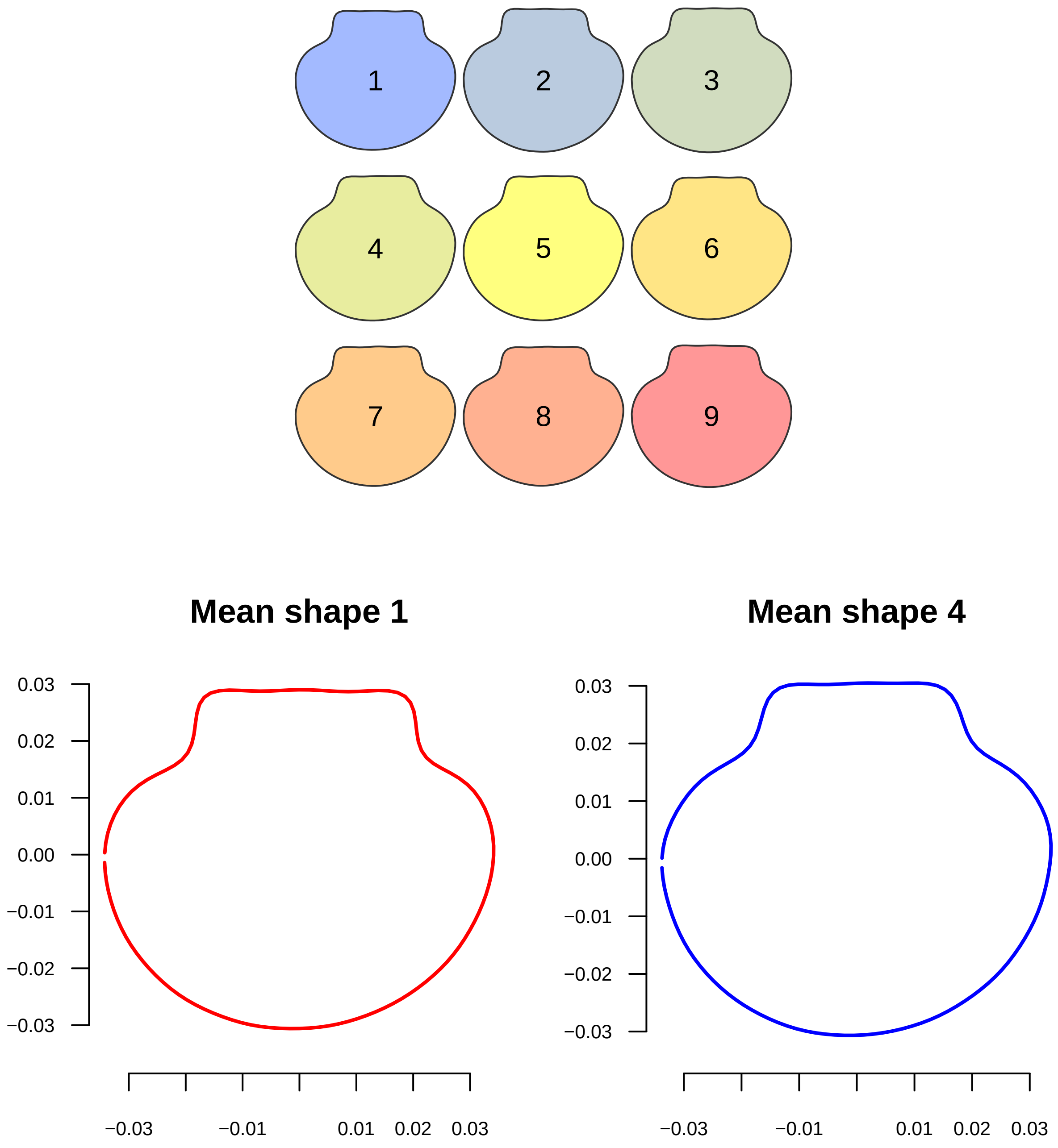

Figure 14-15: Mean shapes for groups (1 to 9) and extreames of the morphospace.

5.1 Thin plate splines analysis (TPS)

Deformation grids first proposed by D'Arcy Thompson can be obtained using the **Thin Plate Splines** (**TPS**) mathematical formalization. TPS analysis is based on the use of harmonic coefficients and can be used to visualise the **deformations** require to pass from the mean shape to the extreme shapes of a morphospace, or to describe mean shape differences among different groups.

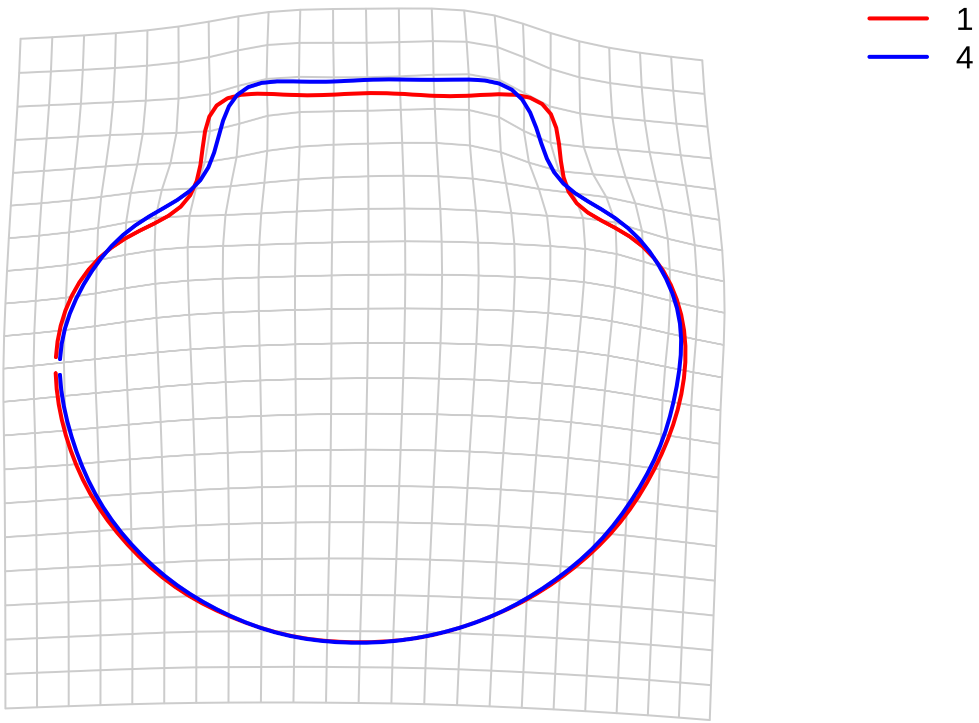

Figure 16: Deformation grid representing the bindings required for passing from the average population 1 shape to 4.

Thin plate splines analysis showed that the main deformations required to pass from the 1 mean shapes to the 4 mean shape were in the hinge area (hinge "shape", height and length). The population 1 was characterised by a more "squared", shorter (height) and longer hinge than 4 that was more "pointy", taller (height) and shorter.

The differences between the two mean shapes were characterized by a slight postero-antero asymmetry of the auricles and adjacent transition from the hinge to the rounder shell outline. Specifically, the right side in 1 was slightly shorter and with a more acute angle than the left one, while for 4 was characterized by marked asymmetries.

As a result, population 4 mean shape resulted more "elongated" than 1, which was slightly tilted towards the right (anterior side).

Data analysis of the shell colour variability (inner left valve) of *Pecten maximus* among nine populations collected along the Northern Ireland coastline is reported.

6 Colour anlysis: data description

Colour data of the inner left valve surface from nine *P. maximus* populations collected along the Northern Ireland coastline (9 *sites*, n=180).

Sample	Site	Area.255	Mean.255	Int.Den.255	Area.110	Mean.110	Int.Den.110	X.Area.110
1	1	9011	132.02	1189600	1794	85.17	152827	0.199
2	1	7525	136.94	1030509	1213	79.77	96784	0.161
3	1	7202	140.25	1010065	806	81.31	65541	0.112
4	1	9530	104.90	999690	5583	76.45	426821	0.586

6.0.1 Variables description

Sample / Site Sample number and scallop population of origin (nine populations from 1 to 9).

Area.255 / Area.110 valve total surface area (grey value from 0 to 255) and coloured surface area (from 0 to 110) in square pixels.

Mean.255 / Mean.110 average pixels grey value for the whole valve and coloured area. This is the sum of the grey values of all the pixels in the selection (respectively grey values 0-255 and 0-110) divided by the number of pixels. These values represent the measure of the pigment intensity for the whole valve surface and the brown coloured area only.

Int.Den.255 / Int.Den.110 respectively the product of $Area.255 \times Mean.255$, and $Area.110 \times Mean.110$.

X.Area.110 proportion of coloured surface area over the total valve area: $\frac{Area.110}{Area.255}$

7 Descriptive statistics

Scallops were characterized by marked *inter* and *intra*-population differences in the colour of the inner flat (left) valve. This brown colour was characterized by a strong variability in both the colour intensity and surface area. The variability of the **amount** of brown colour could be described through the variability of the mean intensity and the proportion of coloured area:

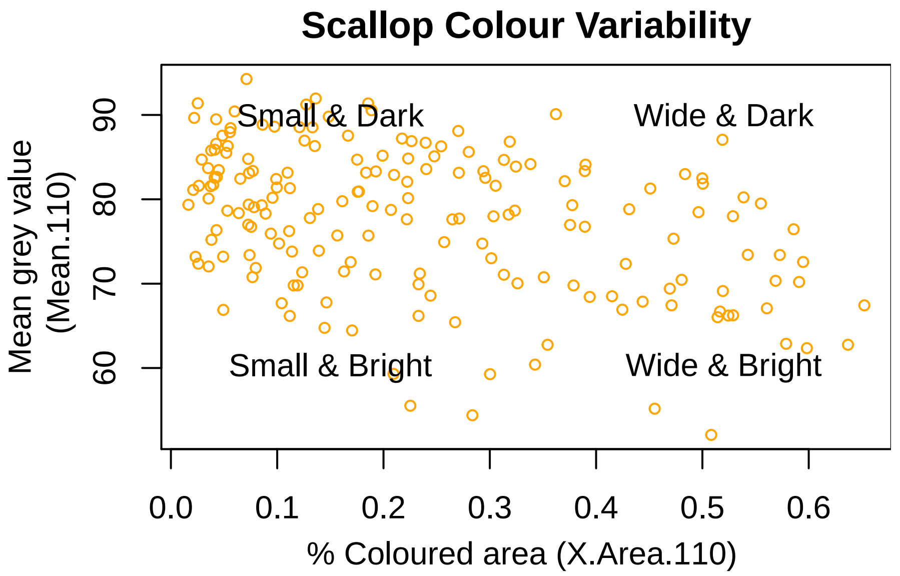

Figure 1: Proportion of coloured area (small / wide) and mean colour intensity (dark / bright) variability for each individual.

Four general groups can be identified, based on the proportion of coloured area and the colour intensity:

1. Individuals with a small and bright coloured area.
2. Individuals with a small and dark coloured area.
3. Individuals with an wide and bright coloured area.
4. Individuals with an wide and dark coloured area.

These two measurements (mean colour intensity and proportion of coloured area) can be used to create an index to **quantify** the brown colour for a given colour area.
The **Mean** $\times$ **Area Index** quantifies the brown coloured area for a given colour intensity and surface area.

```
# Creating a new index to compare the colour variability among  populations
scallop.colour$Mean.X.Area.110 <- scallop.colour$Mean.110 * scallop.colour$X.Area.110
```

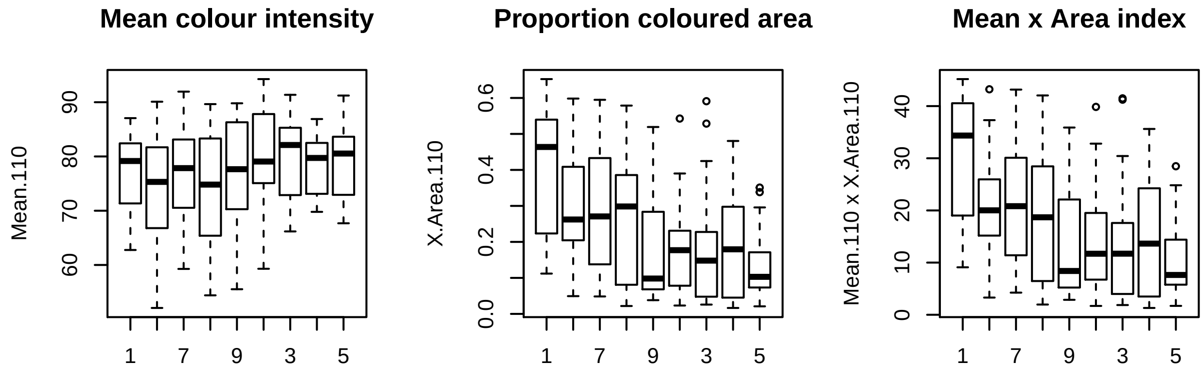

Figure 2: Box plots of **Mean.110**, **X.Area.110* and** Mean.X.Area.110 Index** for the collection sites. The **Mean** $\times$ **Area Index** differed among the nine scallops population:

7.1 Data normality and homoscedasticity

The data were checked for normality and homoscedasticity with histograms, box-plot and Q-Q plot.

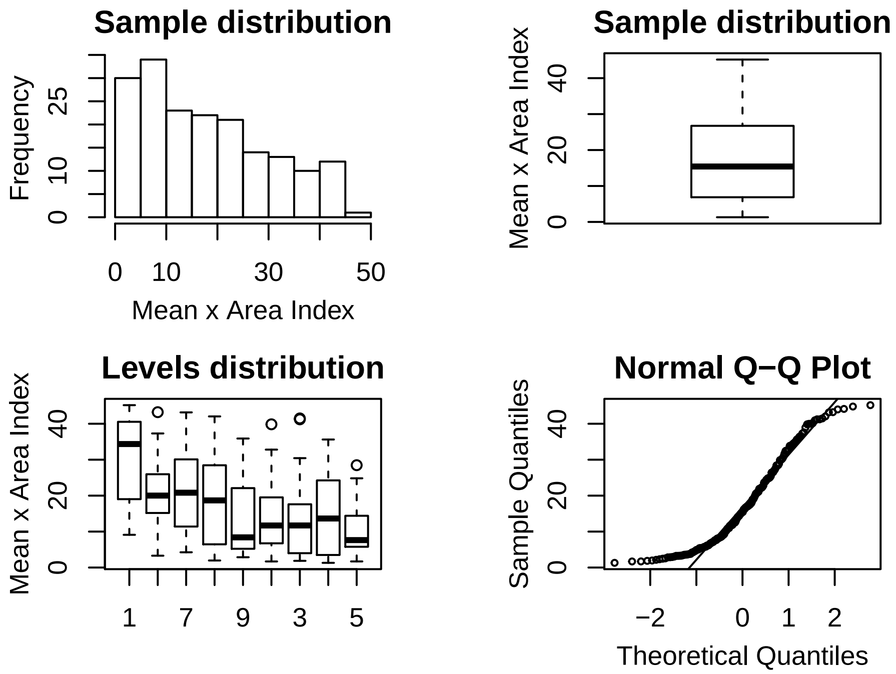

The index was shown to be not normally distributed with a strong positive skew. A square-root transformation was applied in order to make the data more normally distributed and the variances more homogeneous.

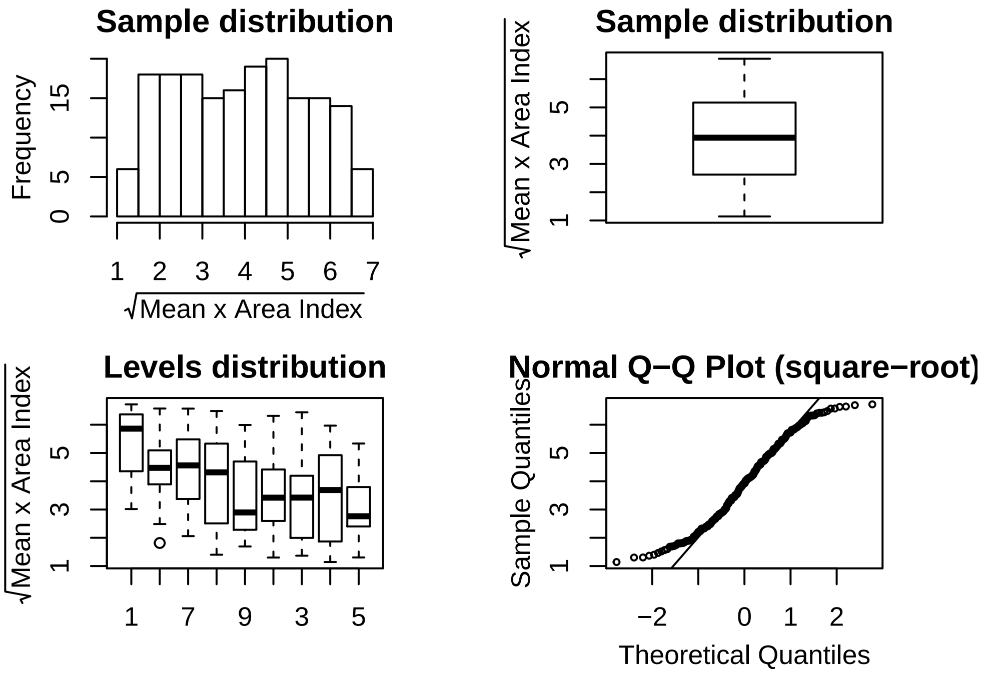

Figure 3: Checking data normality before and after the square-root transformation.

The data distribution was also checked for symmetry before and after the transformation.

```
# Checking distribution simmetry (relative mean and median)
library("boot")
# non-transformed data
median(scallop.colour$Mean.X.Area.110); mean(scallop.colour$Mean.X.Area.110)
```

```
## [1] 15.42354
## [1] 17.83834
```

Before the transformation, the samples median and mean were respectively 15.4 and 17.84, showing a negatively skewed distribution (median higher than mean).

```
# Square-root transformed data
median(sqrt(scallop.colour$Mean.X.Area.110)); mean(sqrt(scallop.colour$Mean.X.Area.110))
```

```
## [1] 3.927281
## [1] 3.936383
```

After a square-root transformation, the median and mean were 3.93 and 3.94, showing a more symmetric, therefore a more normal, distribution.

8 Colour Index

$$Colour\ Index = \sqrt{Mean\ intensity \times Coloured\ area\ (\%)}$$

The **Colour Index** (CI) quantifies the colour on a shell surface for a given colour intensity and surface area. This is used to express the variability of the brown colour of the inner scallop valve.

An high value of the index indicates individuals with a wide and dark coloured area, small values refers to small and brightly coloured shells, while intermediate values indicates intermediate combinations of intensity and area, such as a small and dark or a wide and bright coloured surface (see graph above).

```
# Creation of the the "Colour Index"
scallop.colour$Colour.Index <- sqrt(scallop.colour$Mean.X.Area.110)
```

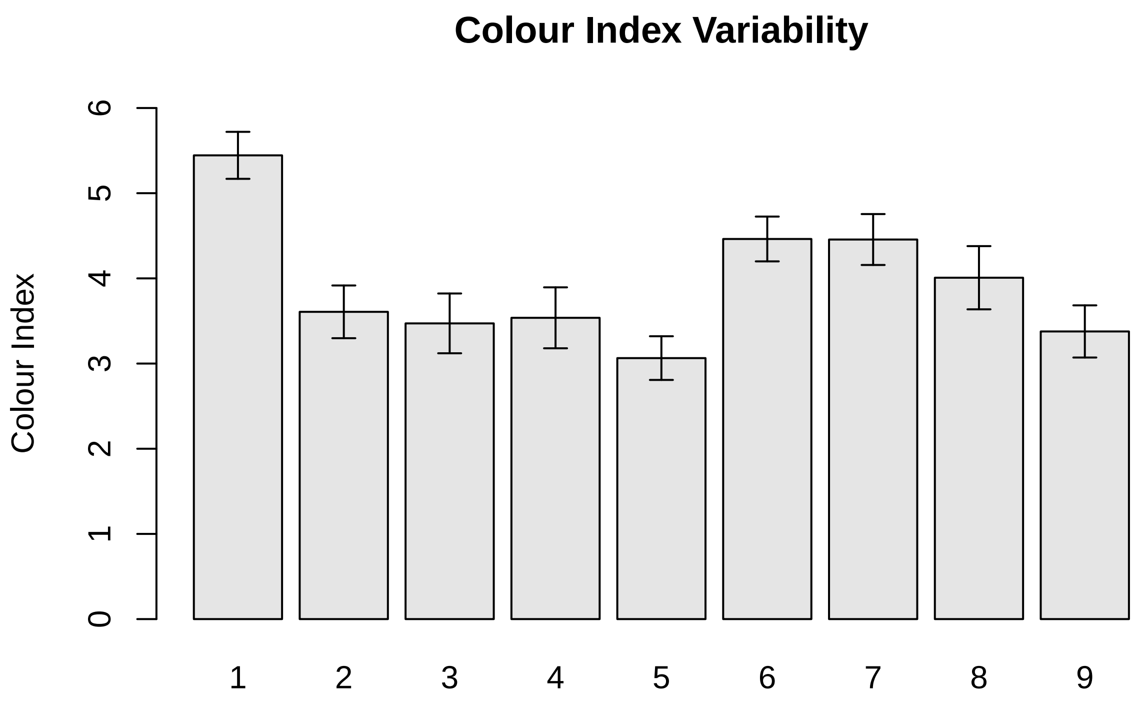

Figure 4: Variability of the Colour Index among the nine scallop populations.

8.1 One-way ANOVA

Hypothesis *"There is a difference in the Colour Index among the nine scallop populations 1-9."*

The data were analysed with a **one-factor Analysis of Variance** in order to test if there was a significant difference of the Colour Index among the nine scallop populations, with and without population 1.

```
# One-way ANOVA colour
colour.aov <- aov(Colour.Index ~ Site, data = scallop.colour)
summary(colour.aov)
```

```
##              Df Sum Sq Mean Sq F value   Pr(>F)    
## Site          8   87.7  10.960    5.61 2.55e-06 ***
## Residuals   171  334.1   1.954                     
## ---
## Signif. codes:  0 '***' 0.001 '**' 0.01 '*' 0.05 '.' 0.1 ' ' 1
```

A **one-way ANOVA** showed a significant difference of the Colour Index among the nine populations ($F_{8,171} = 8.25, p < 0.001, n = 180$).

```r
# One-way ANOVA colour
colour.aov <- aov(scallop.colour$Colour.Index ~ scallop.colour$Site, subset = (Site != "1") )
summary(colour.aov)
```

```
##                      Df Sum Sq Mean Sq F value Pr(>F)
## scallop.colour$Site   7  36.52   5.217   2.598 0.0147 *
## Residuals           152 305.18   2.008
## ---
## Signif. codes:  0 '***' 0.001 '**' 0.01 '*' 0.05 '.' 0.1 ' ' 1
```

A **one-way ANOVA** still showed a significant difference of the Colour Index without population 1 ($F_{7,152} = 2.59, p = 0.015, n = 170$).

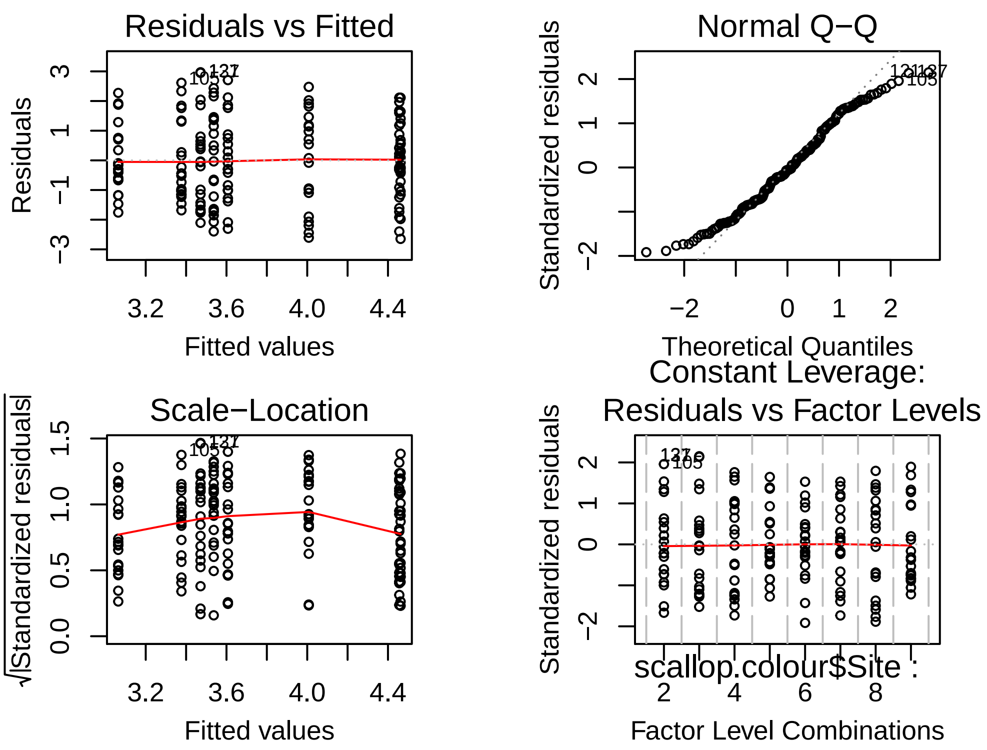

Figure 5: Checking assumptions of residuals normality, homoscedasticity and absence of biased observations.

The one-way ANOVA model met the model assumptions of normality, homogeneity of variance and absence of excessively influential observations.

9 Colour intensity and area

Analysis of the variability of the mean colour intensity and colour surface area among the nine scallop populations. As already defined above.

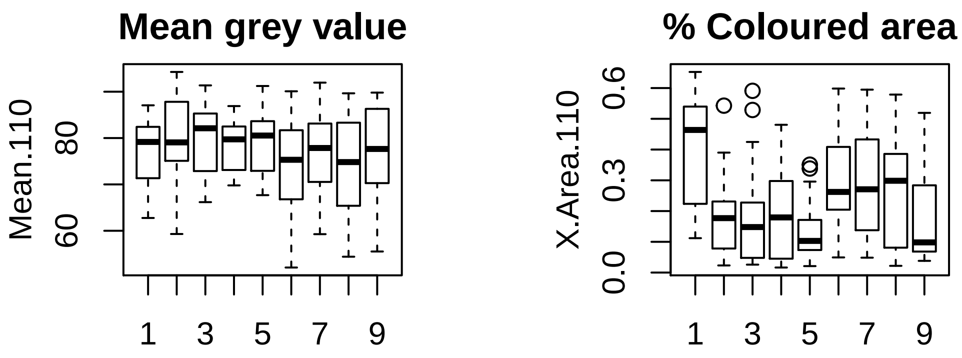

Figure 6 Box plots of the mean grey value and the proportion of the coloured area.

9.1 Colour intensity analysis

Hypothesis *"There is a difference in the mean colour intensity among different populations."*

The data were checked for normality with histograms, box-plot and Q-Q plot; and homogeneity of variance through the analysis of the ratio highest/smallest variance.

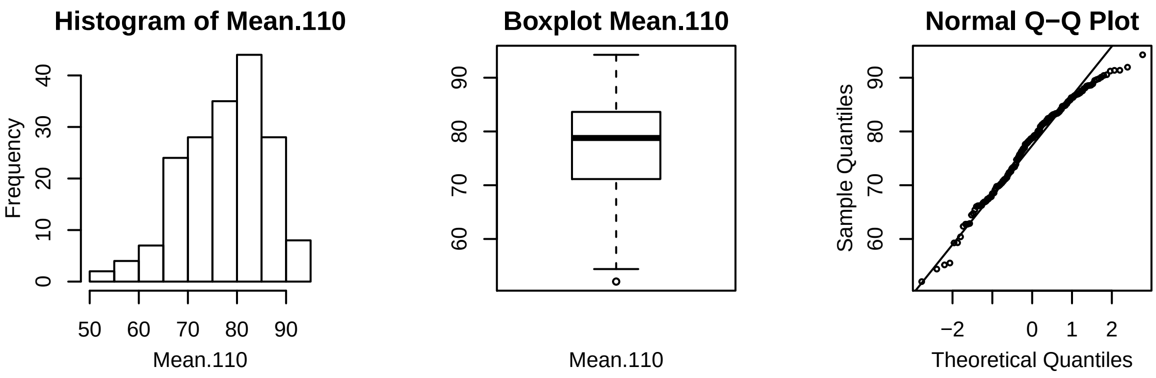

Figure 7: : Checking assumptions of std. residuals normality, homoscedasticity and absence of biased observations.

The colour intensity data were not normally distributed and they were characterized by a strong negative skew. The variances also looked heterogeneus.
In order to make the data distribution more normal the effect of $\log_{10}$, square-root and inverse transformations were checked.

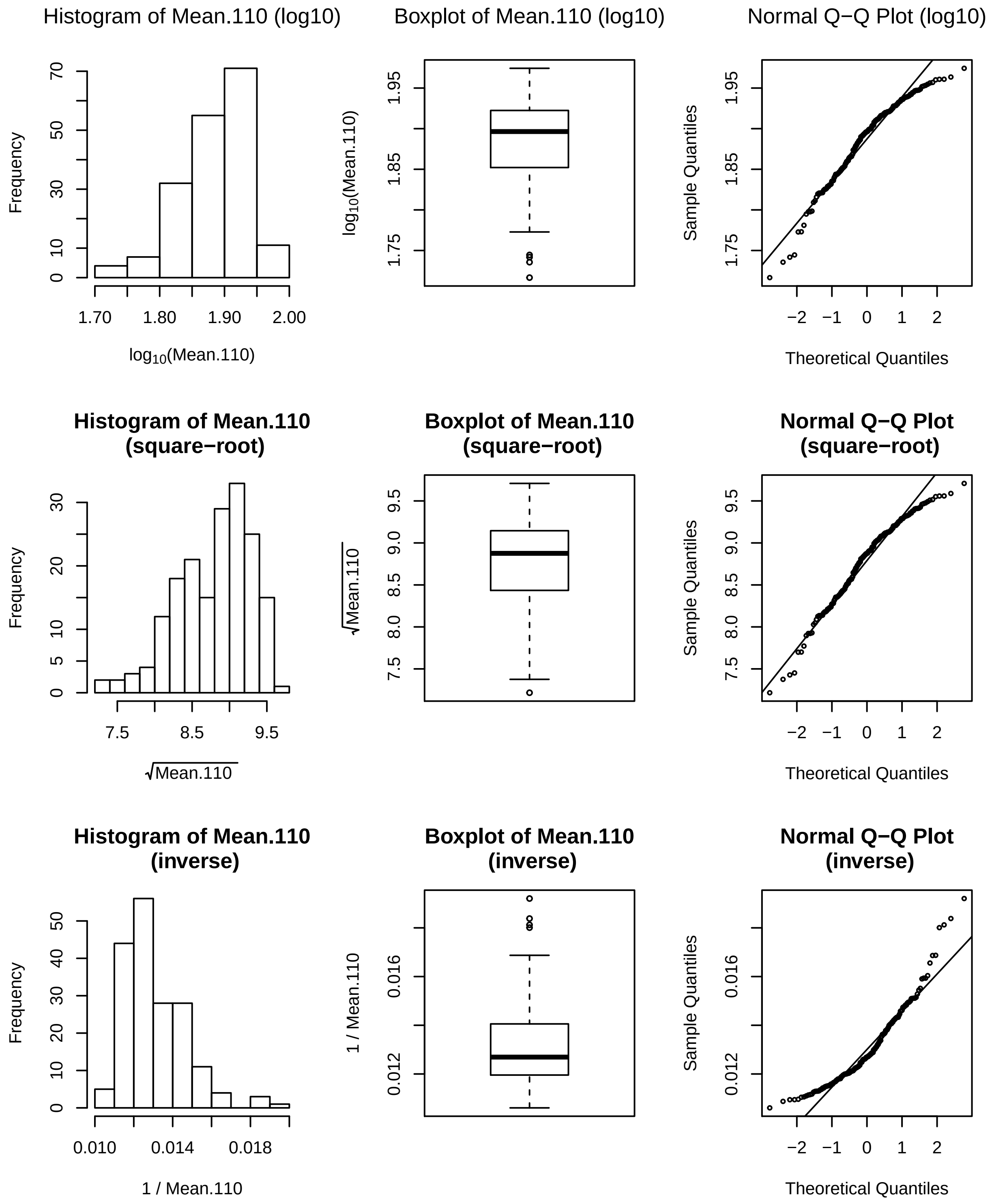

Figure 8: Checking assumptions of std. residuals normality, homoscedasticity and absence of biased observations for the three different transformations.

A $\log_{10}$, square-root and inverse transformations did not make the data distribution more normal. Therefore the variability of the mean grey value among the nine sites was analysed with a **Kruskall-Wallis rank sum Test** (a non-parametric test for multiple independent samples).

```
# Kruskall-Wallis rank  sum Test Mean.110
# non-parametric comparison among three or more independant samples
kruskal.test(Mean.110, Site, data = scallop.colour)
```

```
##
##  Kruskal-Wallis rank sum test
##
## data:  Mean.110 and Site
## Kruskal-Wallis chi-squared = 9.5007, df = 8, p-value = 0.3018
```

A **Kruskall-Wallis rank sum Test** showed that there was a non-significant difference in the mean grey value among the nine scallop populations ($\chi^2_{8,171} = 9.5,\ p = 0.302,\ n = 180$).

9.2 Colour area data analysis

Hypothesis *"There is a difference in the colour surface area among different populations."*

The data were checked for normality with histograms, box-plot and Q-Q plot; and homogeneity of variance through the analysis of the ratio highest/smallest variance.

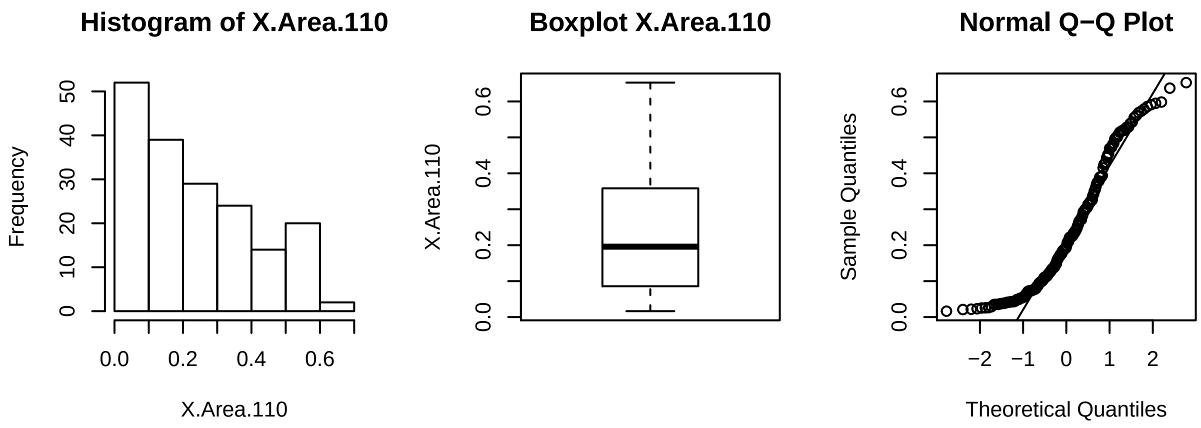

Figure 9: Checking assumptions of std. residuals normality, homoscedasticity and absence of biased observations..

The colour intensity data were not normally distributed and they were characterized by a positive skew. The variances looked heterogeneus.
In order to make the proportional data distribution more normal a arcsin transformation was used.

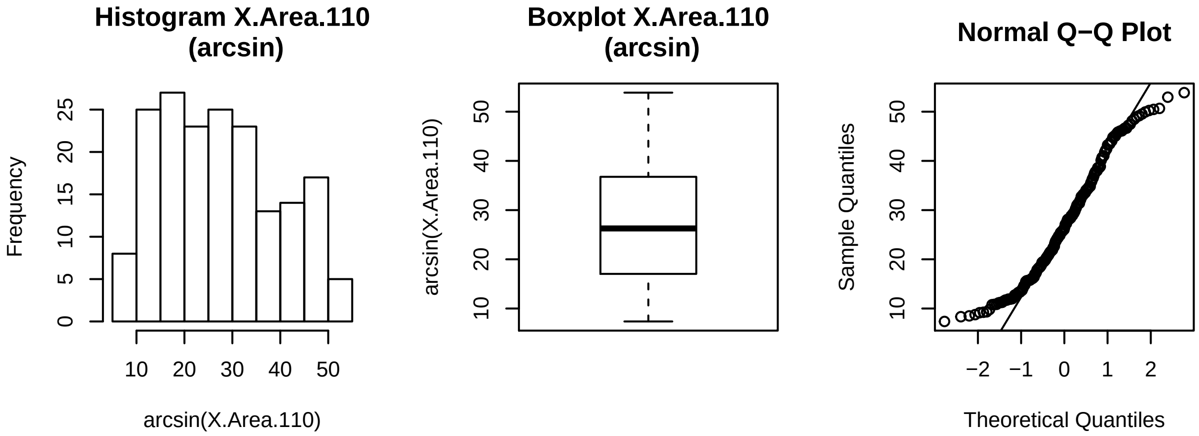

Figure 10: Checking assumptions of std. residuals normality, homoscedasticity and absence of biased observations after an arcsin transformation.

A arcsin transformation made the data distribution more normal, therefore a parametric test was used.

The data were analysed with a **one-factor ANOVA** in order to test if there was a significant difference of the colour surface area among the nine scallop populations.

```
# arcsin transfromation
scallop.colour$arcsin.X.Area.110 <- 57.295*asin(sqrt(X.Area.110))
# One-way ANOVA X.Area.110
X.Area.110.aov <- aov(arcsin.X.Area.110 ~ Site, data = scallop.colour)
summary(X.Area.110.aov)
```

```
##              Df Sum Sq Mean Sq F value   Pr(>F)    
## Site          8   5672   709.0    5.74 1.78e-06 ***
## Residuals   171  21122   123.5                     
## ---
## Signif. codes:  0 '***' 0.001 '**' 0.01 '*' 0.05 '.' 0.1 ' ' 1
```

A **one-way ANOVA** showed a significant difference of the colour surface area (arcsin) among the nine sites ($F_{8,171} = 5.74,\ p < 0.001,\ n = 180$).

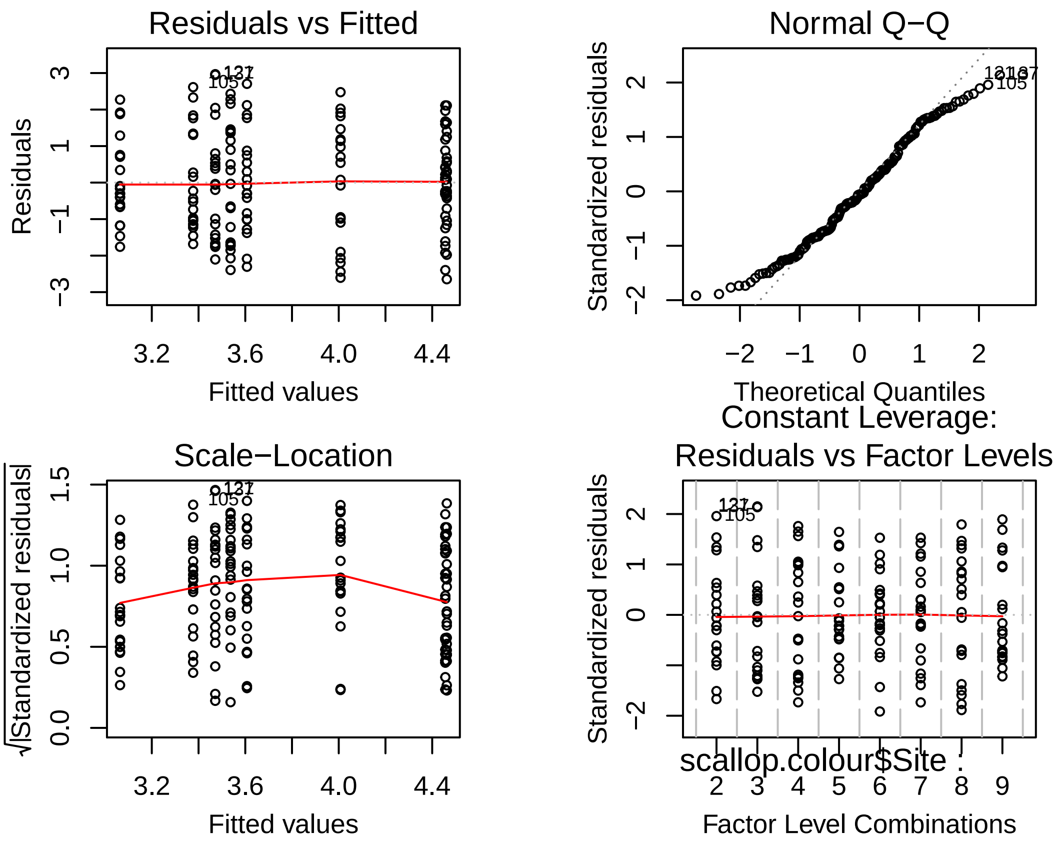

The ANOVA model met the model assumptions of normality, homogeneity of variance and absence of excessively influential observations.

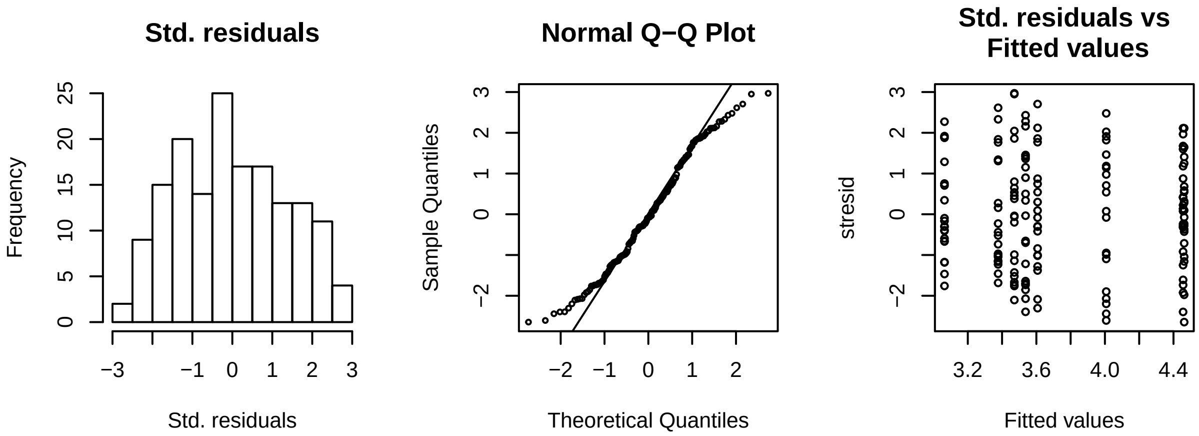

Figure 11-12: Checking assumptions of residuals and std. residuals normality, homoscedasticity and absence of biased observations.

The standardised residuals (error) were normally distributed and homoscedastic.

10 Conclusions (Colour analysis)

A **one-way ANOVA** showed a significant difference of the Colour Index among the nine populations ($F_{8,171} = 8.25, p < 0.001, n = 180$) and without population 1 ($F_{7,152} = 2.59, p = 0.015, n = 170$).

A **Kruskall-Wallis rank sum Test** showed that there was a non-significant difference in the mean grey value among the nine scallop populations ($\chi^2_{8,171} = 9.5,\ p = 0.302$).

A **one-way ANOVA** showed a significant difference of the colour surface area (arcsin) among the nine sites ($F_{8,171} = 5.74,\ p < 0.001$).

There was a significant difference in the Colour Index, of the inner flat valve, among the nine scallop populations (1-9) (**one-way ANOVA**, $F_{8,171} = 8.25, p < 0.001, n = 180$).
This difference in the Colour Index (amount of brown colour on the shell surface) could be mainly explained by a significant difference of the colour surface area among the populations (**one-way ANOVA**, $F_{8,171} = 5.74,\ p < 0.001$), rather than by a real difference in the mean colour intensity (**Kruskall-Wallis rank sum Test**, $\chi^2_{8,171} = 9.5,\ p = 0.302$). Therefore, the differences among the nine populations were caused by a difference in the proportion of the brown-coloured surface area and not by a real difference in the mean colour intensity.
